# InSeq analysis of defined *Legionella pneumophila* libraries identifies a transporter-encoding gene cluster important for intracellular replication in mammalian hosts

**DOI:** 10.1128/mbio.01955-24

**Published:** 2024-10-04

**Authors:** Caitlin E. Moss, Craig R. Roy

**Affiliations:** 1 Department of Microbial Pathogenesis, Yale University, New Haven, Connecticut, USA; 2 Department of Immunobiology, Yale University, New Haven, Connecticut, USA; Institut Pasteur, Paris, France

**Keywords:** intracellular bacteria, protein secretion, ABC transporters, *Legionella pneumophila*, transposon mutant sequencing

## Abstract

*Legionella pneumophila* is an intracellular bacterial pathogen that replicates inside human alveolar macrophages to cause a severe pneumonia known as Legionnaires’ disease. *L. pneumophila* requires the Dot/Icm Type IV secretion system to deliver hundreds of bacterial proteins to the host cytosol that manipulate cellular processes to establish a protected compartment for bacterial replication known as the *Legionella-*containing vacuole. To better understand mechanisms apart from the Dot/Icm system that support survival and replication in this vacuole, we used transposon insertion sequencing in combination with defined mutant sublibraries to identify *L. pneumophila* fitness determinants in primary mouse macrophages and the mouse lung. This approach validated that many previously identified genes important for intracellular replication were critical for infection of a mammalian host. Further, the screens uncovered additional genes contributing to *L. pneumophila* replication in mammalian infection models. This included a cluster of seven genes in which insertion mutations resulted in *L. pneumophila* fitness defects in mammalian hosts. Generation of isogenic deletion mutants and genetic complementation studies verified the importance of genes within this locus for infection of mammalian cells. Genes in this cluster are predicted to encode nucleotide-modifying enzymes, a protein of unknown function, and an atypical ATP-binding cassette (ABC) transporter with significant homology to multidrug efflux pumps that has been named Lit*,* for *Legionella* infectivity transporter. Overall, these data provide a comprehensive overview of the bacterial processes that support *L. pneumophila* replication in a mammalian host and offer insight into the unique challenges posed by the intravacuolar environment.

**IMPORTANCE:**

Intracellular bacteria employ diverse mechanisms to survive and replicate inside the inhospitable environment of host cells. *Legionella pneumophila* is an opportunistic human pathogen and a model system for studying intracellular host–pathogen interactions. Transposon sequencing is an invaluable tool for identifying bacterial genes contributing to infection, but current animal models for *L. pneumophila* are suboptimal for conventional screens using saturated mutant libraries. This study employed a series of defined transposon mutant libraries to identify determinants of *L. pneumophila* fitness in mammalian hosts, which include a newly identified bacterial transporter called Lit. Understanding the requirements for survival and replication inside host cells informs us about the environment bacteria encounter during infection and the mechanisms they employ to make this environment habitable. Such knowledge will be key to addressing future challenges in treating infections caused by intracellular bacteria.

## INTRODUCTION

The Gram-negative intracellular bacterial pathogen *Legionella pneumophila* is a master of host cell manipulation (1, 2). *L. pneumophila* is a natural parasite of freshwater protozoa with a vast arsenal of molecular tools allowing it to infect an incredibly wide range of species (3). The strategies that *L. pneumophila* evolved to manipulate host processes in diverse amoebae also confer an ability to replicate within human alveolar macrophages, which can result in a severe pneumonia called Legionnaires’ disease (4, 5). Upon internalization by a host cell, *L. pneumophila* uses the Dot/Icm Type IV secretion system to deliver over 300 different bacterial proteins across the phagosomal membrane into the host cytosol (6, 7). The collective action of these secreted proteins, known as effectors, allows *L. pneumophila* to prevent vacuole acidification, recruit ER-derived vesicles, and persist in a protected compartment called the *Legionella-*containing vacuole (LCV) (8–11). A functional Dot/Icm machine is required for intracellular replication, but most individual effectors, and even clusters of effectors, can be inactivated without measurable disruption of the infection cycle, making it a challenge to decipher the contributions of each effector (12–14).

In addition to effector delivery via the Dot/Icm system, Dot/Icm-independent factors contribute to *L. pneumophila* fitness in a host cell. Notably, there are several examples of membrane-bound transporters that support *L. pneumophila* intracellular survival and replication. It has been demonstrated that a Type II secretion apparatus is required for optimal replication in amoebae, human macrophages, mice, and guinea pigs (15–17). Over 20 substrates of the *L. pneumophila* Type II system are known, some of which are themselves critical for infection of protozoa (18, 19). Many of these substrates are involved in catabolism of proteins, peptides, and complex carbohydrates, indicating that this system may contribute to nutritional support of *L. pneumophila* in the LCV (20). Components of less-complex transporters also support *L. pneumophila* infection. For example, mutants deficient in the outer-membrane channel TolC are severely disadvantaged during infection of protozoan and macrophage-like hosts (21). TolC confers resistance to diverse antimicrobial agents via efflux; however, no physiologically relevant substrates in *L. pneumophila* have been identified. Additionally, although amino acids are a crucial source of energy for *L. pneumophila* during infection, surprisingly little is known about specific mechanisms for nutrient uptake. *L. pneumophila* is auxotrophic for seven amino acids (22), but only a threonine transporter, required for infection of murine bone marrow-derived macrophages (BMDMs), has been characterized (23). Ostensibly, mechanisms like these precisely modulate import and export across the bacterial membranes, allowing access to necessary resources while preventing a buildup of toxic compounds of bacterial or host origin. Still, the genetic requirements for *L. pneumophila* to overcome the challenges of surviving and replicating within the LCV remain poorly understood.

It is widely appreciated that transposon (Tn) insertion sequencing approaches (InSeq, TnSeq), are invaluable for identifying conditionally essential bacterial genes in a high-throughput manner (24–27). This method has been applied in *L. pneumophila* to elucidate requirements for infection; however, large-scale genetic screens of this kind have focused primarily on protozoan hosts (28–30). Therefore, mechanisms supporting *L. pneumophila* fitness within a mammalian host cell have not been subject to systematic analysis.

The limitations of mammalian infection models for *L. pneumophila* complicate high-throughput screens due to the stochastic effects that can result from population bottlenecks. Our laboratory has shown that bottlenecks can be avoided by using low-complexity Tn libraries for screens in mammalian hosts (31). In this previous study, an arrayed Tn mutant library was generated from which 528 independent Dot/Icm effector mutants were isolated. Pooling these mutants into a defined library for InSeq analysis enabled the discovery of novel effector phenotypes by enhancing the screen resolution to reveal subtle fitness changes (31). Here, we expanded this approach by generating defined sublibraries, each containing fewer than 1,000 mutants, from the existing arrayed library, with the goal of identifying *L. pneumophila* fitness determinants in mammalian hosts. Mutants with reduced fitness in mouse lungs and BMDMs were identified, which include mutants deficient in known virulence factors and a collection of newly-identified genes important for replication in mammalian cells.

## RESULTS

### Screening of *L. pneumophila* transposon mutant sublibraries for fitness defects in mice and primary macrophages

An arrayed library of 10,163 independent *L. pneumophila* Tn insertion mutants was screened for fitness defects in BMDMs and mice. To avoid population bottlenecks during infection, we constructed 15 sublibraries, each containing 500–700 different mutants. Sublibraries were created by growing individual mutants spotted onto solid media from library plates and subsequently combining mutants into pools (Fig. S1A and B). Growth was largely consistent across mutants, but instances where mutants showed little or no growth were noted. Importantly, because InSeq analysis measures relative changes in bacterial population, it was unnecessary to have equal amounts of each mutant in the sublibraries (31, 32). Sequencing confirmed that the sublibraries contained the expected mutants based on previous mapping of the arrayed library (31) (Table S1). Although some expected mutants were not detected, many of these corresponded to mutants that were not recovered during sublibrary construction. The composition of sublibraries cultured from single-use aliquots was highly reproducible across experiments (Fig. S1C). However, we observed stochastic differences in abundance for poorly represented mutants, prompting us to exclude mutants with input reads fewer than five counts per million during analysis. Each sublibrary contained at least five *dot/icm* insertion mutants, which have strong intracellular replication defects, to serve as internal controls during screening.

To define genetic requirements for *L. pneumophila* fitness in mammalian hosts, we used each of the 15 Tn sublibraries to infect A/J BMDMs *ex vivo* and A/J mice intranasally (Fig. S2). BMDM infections were performed at a multiplicity of infection (MOI) of 0.1, and the mouse inoculum of 5 × 10^5^ bacteria was chosen to ensure expansion of the population throughout infection (Fig. S3). In both screens, output populations were collected after 48 h of infection for InSeq analysis. Additionally, we performed a control screen by passaging sublibraries on charcoal–yeast extract (CYE) agar, the standard media for *L. pneumophila* axenic replication (Fig. S2). After 72 h of growth, the resulting output populations were compared to the inputs to identify mutants with generalized growth defects on CYE, which were reasoned to be unrelated to selection pressures exerted during infection of a host.

### Analysis of bacterial functional categories contributing to *L. pneumophila* fitness

**Fig 1:**
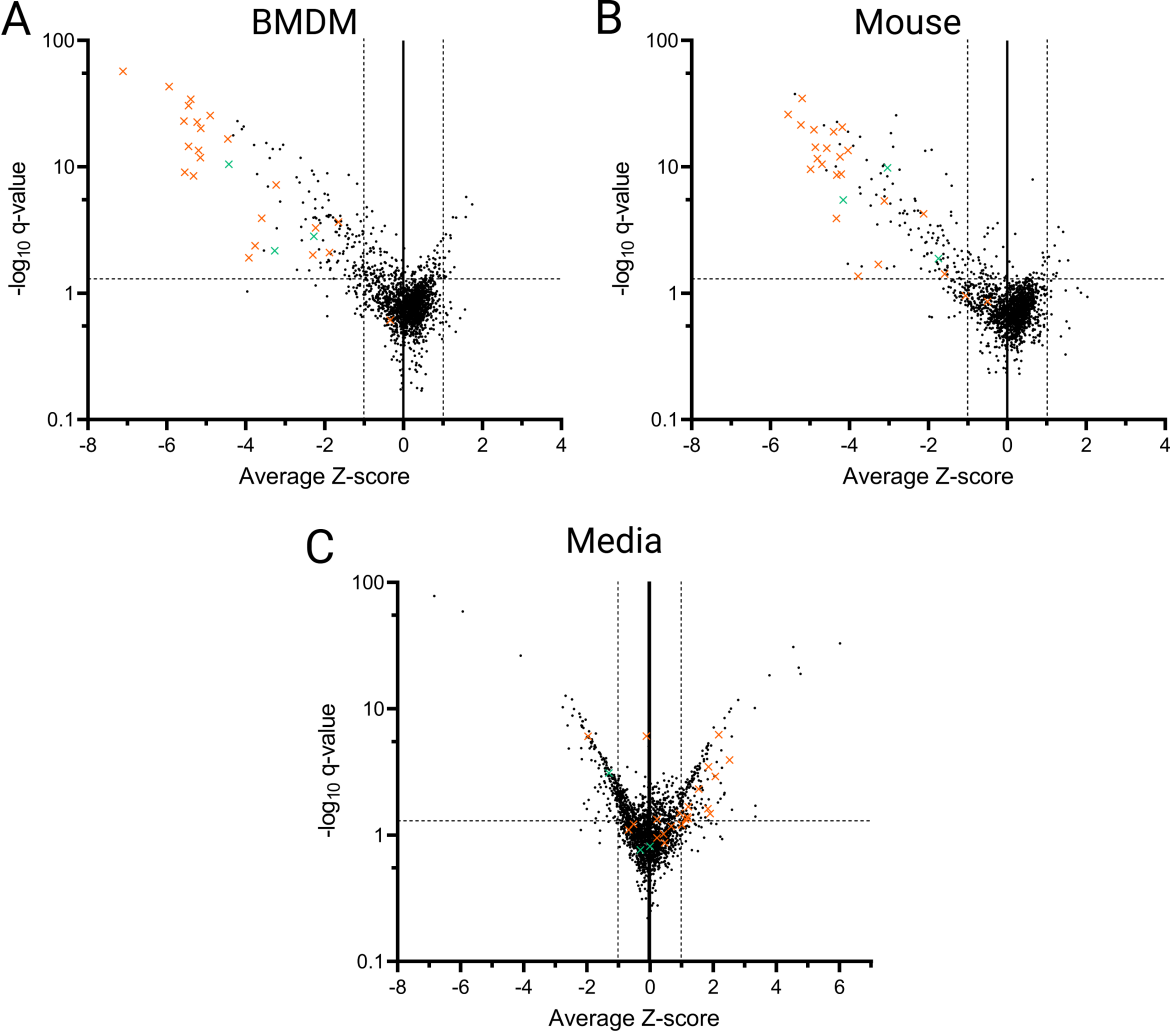
Identification of *L. pneumophila* genes required for optimal fitness. Volcano plots indicate fitness scores (*Z*-scores) of *L. pneumophila* genes during selection in (**A**) A/J mouse BMDMs, (**B**) A/J mouse lungs, and (**C**) standard laboratory media. Each point represents the average *Z*-score and *q*-value of Tn mutants in a single gene. Dotted lines indicate the significance cutoff values of Z > 1 or Z < −1, and *q* < 0.05. Orange Xs indicate *dot/icm* genes, and green Xs indicate the *dot/icm* effector genes *mavN*, *sdhA*, and *mesI*.

For each screen, relative abundances of mutants in the output populations were compared to their respective inputs by InSeq analysis (24, 32). Mutants were designated significantly over- or underrepresented in the output population based on statistical analysis (*q* < 0.05) and a log_10_ output:input ratio greater than 1 standard deviation from the overall population (*Z* > 1 or *Z* < −1) (Fig. S4 to S6; Data Sets S1 to S3). The majority of Tn insertions did not affect bacterial fitness, and these mutants were represented equally well pre- and post-selection (Fig. 1). To identify genes that, when disrupted, resulted in significant fitness defects, we focused on mutants that were underrepresented in the outputs (Z < −1). Many genes were represented by multiple independent mutants contained in different sublibraries; therefore, the effect of these genes on bacterial fitness was tested multiple times. In these cases, we focused on genes for which ≥60% of mutants met the above thresholds (Data Set S4).

**Fig 2:**
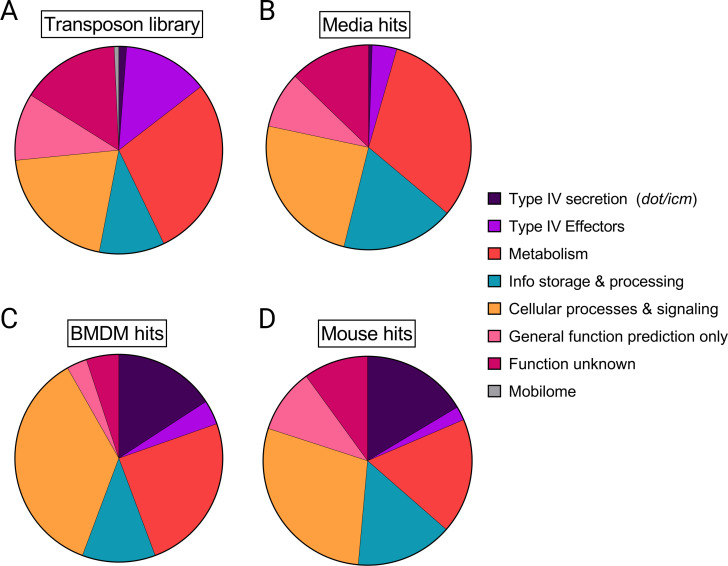
Functional requirements for *L. pneumophila* growth in mammalian hosts differ from those in media and are comprised of both *dot/icm* and non-*dot/icm* genes. Pie charts represent a functional analysis of genes required for optimal growth during the screens. The functional categories include COGs as well as *dot/icm* genes and cognate Type IV effectors. (**A**) The distribution of cellular functions represented by the full panel of mutants in the Tn library pre-selection. (**B through D**) Cellular functions required for optimal growth in on solid laboratory media (**B**), in A/J BMDMs (**C**), and in A/J mice (**D**).

For a broad understanding of cellular functions contributing to *L. pneumophila* fitness under the conditions studied, we examined the Clusters of Orthologous Genes (COG) categories of hits based on existing annotations (33, 34). Relative to the COG distribution of the total Tn library, growth on media led to weak selection for functions in the Metabolism and Info Storage and Processing categories (Fig. 2). Mainly, these functions consisted of energy production/conversion, amino acid transport/metabolism, translation/ribosome structure, and DNA replication/repair.

As expected, *L. pneumophila* with insertions in *dot/icm* genes represented a major subset of the mutants with strong fitness defects in mice and BMDMs (Fig. 2). Notably, although over 10% of mutants in the total library were deficient in Dot/Icm-translocated effectors, very few effector mutants displayed fitness defects, a well-documented phenomenon in *L. pneumophila* (14, 35). Many mutants with decreased fitness during infection carried insertions in genes belonging to the Cellular Processes and Signaling COG, a diverse category including genes important for cell wall structure, intracellular trafficking, secretion, signal transduction, post-translational modification, protein stability/turnover, and virulence mechanisms. Many genes in this COG that contributed to growth in mammalian hosts are predicted to encode transporter components. Some of these are known to support *L. pneumophila* infection in murine BMDMs (*phtA* [23]), or in other hosts, such as amoebae or U-937 human macrophage-like cells (*tolC*, *tatC* [21, 36, 37]), but many remain uncharacterized.

### Identification of genes supporting *L. pneumophila* survival and replication in mammalian hosts

**Fig 3:**
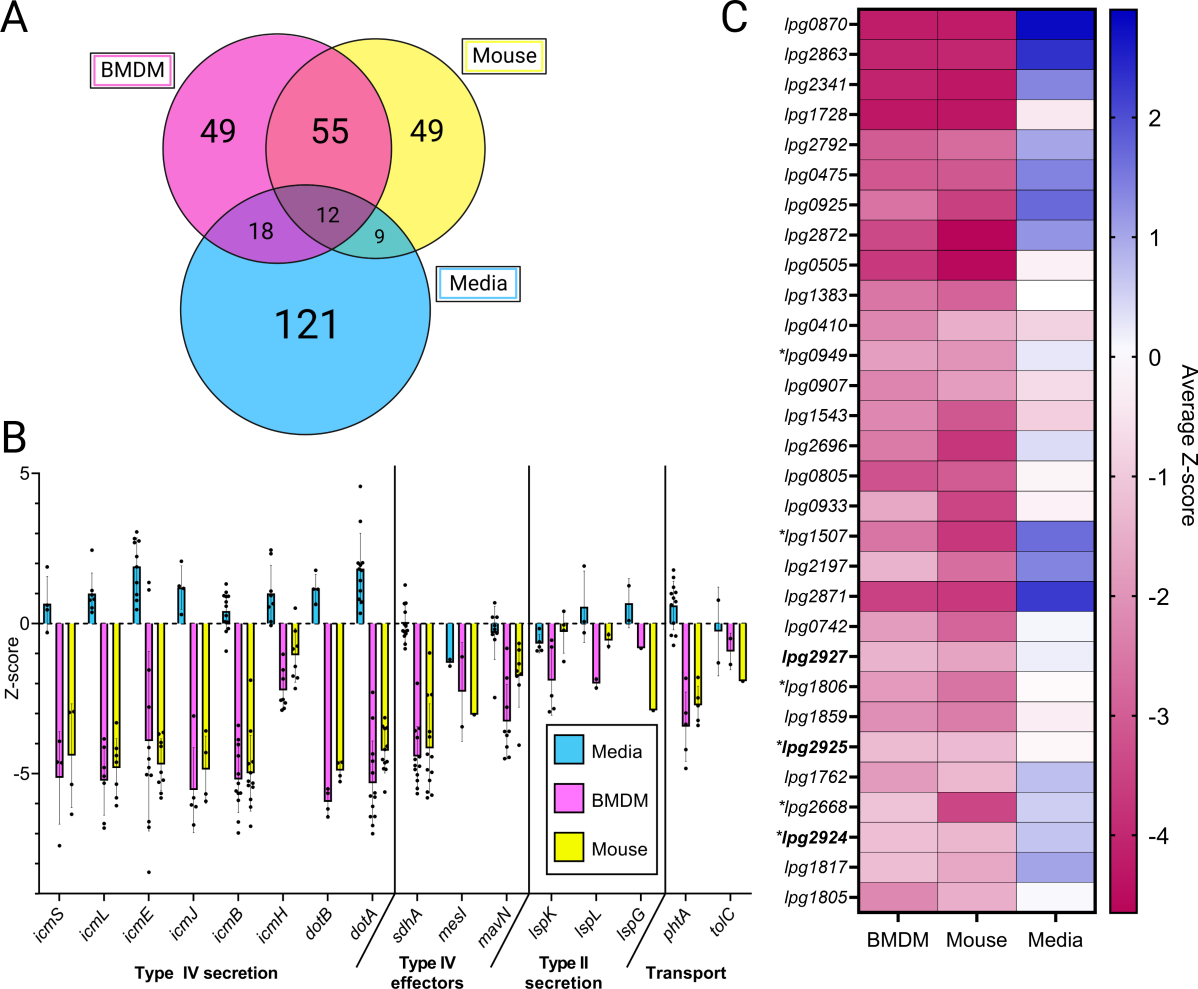
*L. pneumophila* genes contributing to growth in A/J mice and BMDMs include known virulence factors and novel fitness determinants. (**A**) Comparison of genes required for *L. pneumophila* fitness in each screen. (**B**) A selection of fitness data corresponding to mutants in virulence factors with previously demonstrated contributions to bacterial growth in primary macrophages and/or mice. For brevity, eight *dot/icm* genes were chosen at random. For each gene indicated, *Z*-scores of each individual Tn mutant in the gene are plotted (dots), and the mean *Z*-score is shown (bars). (**C**) Heat map indicating the average Z-score of Tn mutants in a given gene, where magenta specifies mutants that were underrepresented in a screen (genes are required for fitness), and blue indicates mutants that were overrepresented (gene loss confers an advantage). Each column represents results from one of the three screens. The table is ranked by *q*-values from the BMDM screen, with the most significant results at the top. Genes encoding transporter components are indicated with asterisks. Genes in the cluster selected for further study are indicated in bold. “Known” genes from Table S2 have been omitted. Panel **A **was created with BioRender.com.

A Venn diagram illustrates the overlap of genes in which Tn insertions decreased fitness in media, BMDMs, and mice (Fig. 3A; detailed gene lists in Data Set S5). Mutants in 12 genes displayed fitness defects in all conditions. Most of these commonalities encode proteins involved in transcription, translation, or ribosome structure, including regulators *oxyR*, *dksA*, and *cspA*, and rRNA modifier *rluC* (38–42). In general, we observed little overlap between fitness determinants in the CYE and infection conditions, suggesting that laboratory media for *L. pneumophila* cultivation is poorly representative of the intracellular environment encountered by the bacteria.

In contrast, the overlap between genes required for fitness in BMDMs and mice was greater. We focused on the 55 genes required in both infection models but not media (Fig. 3A). Within this group were *dot/icm* genes and effector genes *sdhA*, *mavN*, and *mesI* (Fig. 3B), mutants of which have known intracellular replication defects (31, 43–45). Transporter genes *phtA* and *tolC* (21, 23, 46, 47) and Type II secretion machinery components (17) were also in this category. Thus, our screens reliably identified genes that are important for infection of mammalian hosts.

Excluding genes with known importance during infection of mammalian cells (Table S2), we identified 30 genes supporting *L. pneumophila* fitness in both BMDMs and mice (Fig. 3C). Most were dispensable for growth on media, and in many cases, gene disruption was favorable for axenic growth. The newly identified genes most required for fitness in both infection models were enoyl-CoA hydratase *lpg0870*, pteridine reductase *lpg2863*, heat shock chaperone *lpg2341*, and hypothetical gene *lpg1728* (Fig. 3C).

### Examination of a gene cluster important for intracellular growth

**Fig 4:**
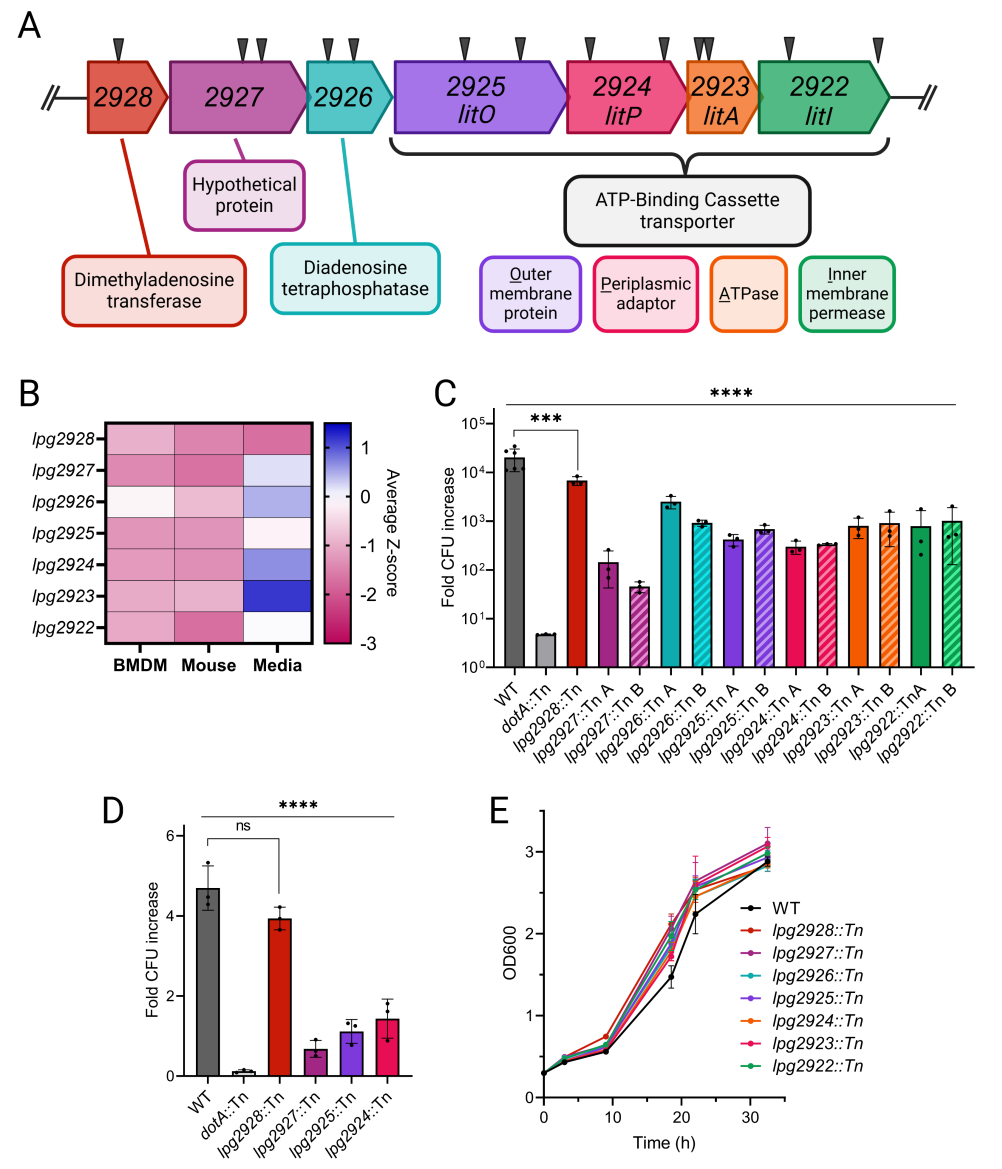
Tn insertion mutants in a seven-gene cluster display decreased fitness in mammalian hosts. (**A**) Locus diagram indicating locations of Tn insertions (black triangles) in the mutants isolated from the arrayed library for validation experiments. Genes are labeled by *lpg* numbers and existing genomic annotations; gene arrowheads indicate direction of transcription. (**B**) Heat map showing the average *Z*-scores of mutants in the genes *lpg2928–lpg2922,* indicating over- or underrepresentation in each screen as detailed in Fig. 3. (**C**) Fold change in colony-forming units (CFUs) of individual Tn mutants in A/J BMDMs over 72 h of infection. Two independent Tn mutants in each gene were used (where available) and are marked A and B. (**D**) Fold change in CFUs of Tn mutants isolated from the lungs of A/J mice over 48 h of infection. (**E**) Growth of Tn mutants in standard liquid media. Asterisks in panels **C and D** indicate statistical significance by one-way analysis of variance (ANOVA) (***P < 0.001, ****P < 0.0001). Results are representative of at least two independent experiments. Panel **A** was created with BioRender.com.

We observed that syntenic genes *lpg2924*, *lpg2925*, and *lpg2927* contributed to fitness in both host environments (Fig. 3C). Additionally, *lpg2922* was identified in the BMDM screen and *lpg2928* in the mouse screen (Fig. S7A and B). These hits define a gene cluster spanning from *lpg2928* to *lpg2922* (Fig. 4A). Further inspection of the InSeq data revealed that mutants with Tn insertions in six of these genes were underrepresented in both host screens. The exception, *lpg2926*, was neutral in BMDMs (Fig. 4B). The largest intergenic gap in the cluster is 43 bp, between *lpg2926* and *lpg2925*, and most of the annotated reading frames overlap slightly. The flanking genes, *lpg2929* and *lpg2921*, have coding regions on the opposite strand. These data suggest that the clustered genes may encode proteins with a shared function in promoting intracellular replication of *L. pneumophila*.

To verify the importance of this gene cluster for *L. pneumophila* replication in BMDMs, we isolated individual Tn mutants for each gene from the arrayed library. If available, two independent insertion mutants were isolated per gene (Fig. 4A). Similar to the screening results, the isolated mutants *lpg2927*::Tn, *lpg2925*::Tn, *lpg2924*::Tn, and *lpg2922*::Tn displayed 20- to 447-fold defects in intracellular replication after 72 h compared to the parental (WT) strain (Fig. 4C). The *lpg2928*::Tn mutant, which was underrepresented in the mouse screen, had a modest yet significant replication defect when measured independently in BMDMs (Fig. 4C). However, this mutant was also underrepresented in the media screen, which may indicate that disruption of *lpg2928* causes an intrinsic replication defect that is not specific to infection. Although the *lpg2923*::Tn and *lpg2926*::Tn mutants were not significantly underrepresented during InSeq analysis, they were significantly defective for replication in BMDMs when tested individually (Fig. 4C). These data indicate that genes within the *lpg2928–lpg2922* region are important for *L. pneumophila* replication in mammalian cells.

To verify that these genes are also important for *L. pneumophila* virulence, A/J mice were intranasally inoculated with individual mutants *lpg2928*::Tn, *lpg2927*::Tn, *lpg2925*::Tn, or *lpg2924*::Tn. The mutants *lpg2927*::Tn, *lpg2925*::Tn, and *lpg2924*::Tn displayed severe growth defects in the mouse lung compared to WT (Fig. 4D). The *lpg2928*::Tn mutant exhibited a slight defect that did not reach statistical significance. Importantly, Tn mutants in the *lpg2928–lpg2922* region grew indistinguishably from the WT strain in broth (Fig. 4E). This includes *lpg2928*::Tn, which was underrepresented in the screen performed on solid media. Overall, we concluded that genes within the *lpg2928–lpg2922* locus are important for intracellular replication in primary macrophages and in the mouse lung.

### Analysis of isogenic deletion mutants delineates the importance of genes in the *lpg2928–lpg2922* cluster for intracellular replication of *L. pneumophila*

**Fig 5:**
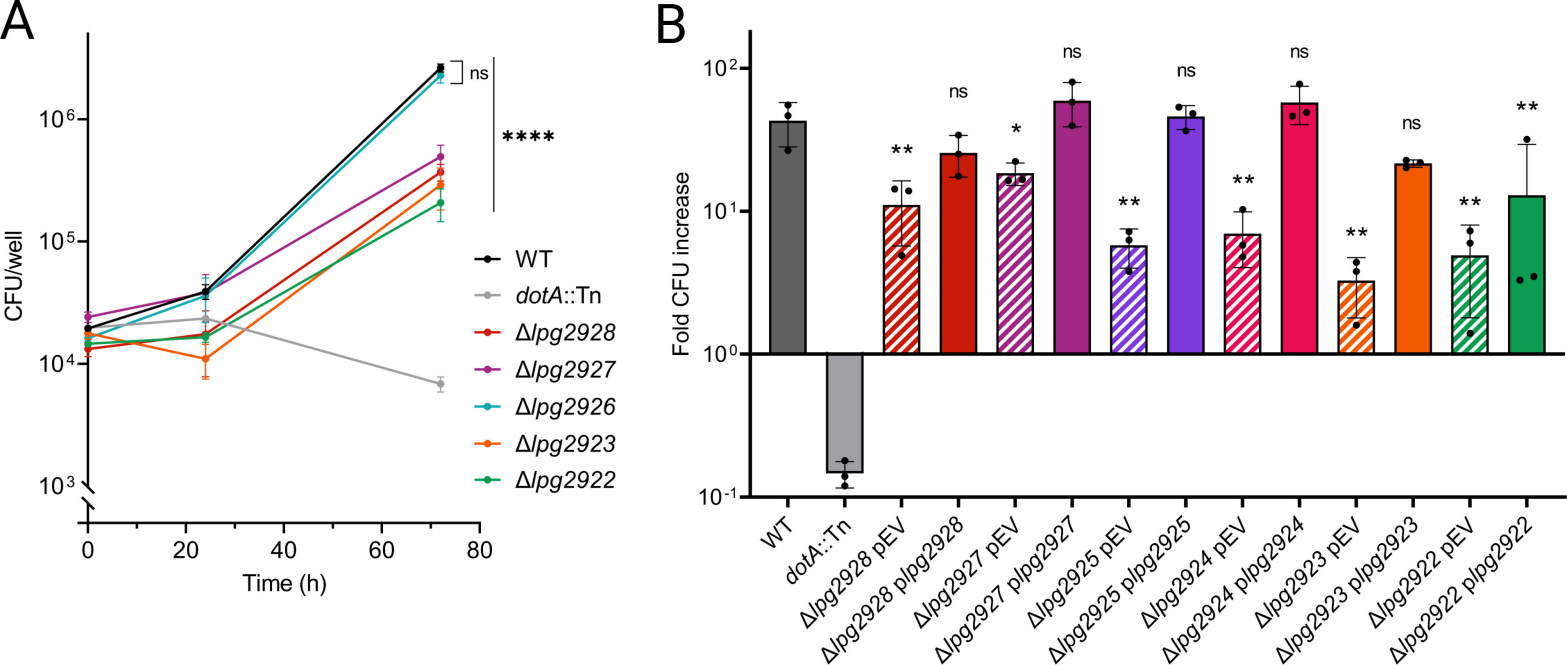
Analysis of in-frame chromosomal deletion strains clarifies the importance of genes in *lpg2928–lpg2922* during BMDM infection. (**A**) Growth of isogenic deletion mutants in A/J BMDMs over 72 h of infection compared to WT *L. pneumophila*. (**B**) Fold change in CFUs of deletion mutants complemented in *trans* via IPTG-inducible plasmids. pEV, empty vector. Asterisks indicate statistical significance by one-way ANOVA relative to WT (*P < 0.05, **P < 0.01, ****P < 0.0001). Results are representative of at least two independent experiments.

Because Tn insertions can have polar effects on the expression of downstream genes, it was difficult to ascertain which genes in the *lpg2928–lpg2922* cluster contribute to *L. pneumophila* intracellular replication based solely on Tn mutant data. Thus, we constructed isogenic mutants containing an in-frame deletion for each gene in the *lpg2928–lpg2922* cluster to define the genes important for infection. Intracellular replication defects were observed for Δ*lpg2928*, Δ*lpg2927*, Δ*lpg2923*, and Δ*lpg2922* mutants, validating that these genes support *L. pneumophila* infection of mammalian hosts (Fig. 5A). No intracellular replication defect was detected for the Δ*lpg2926* mutant, suggesting that the defects of the *lpg2926*::Tn mutant likely resulted from a polar effect interfering with expression of downstream genes.

Complementation studies were conducted using inducible copies of individual genes on plasmids. The replication defects of the Δ*lpg2928*, Δ*lpg2927*, Δ*lpg2925*, Δ*lpg2924*, and Δ*lpg2923* mutants in BMDMs were complemented by supplying the corresponding gene in *trans* (Fig. 5B). The replication defect of the Δ*lpg2922* mutant was partially rescued by plasmid complementation. Because *lpg2922* is predicted to encode an inner membrane protein, it is likely that gene dosage is critical for complementation, and expression from a plasmid resulted in a detrimental overproduction of protein. In all, these data demonstrate that six genes in the *lpg2928–lpg2922* cluster are important for replication of *L. pneumophila* in mammalian cells.

### The genes *lpg2925–lpg2922* are predicted to encode an ABC transporter

To better understand the role of the *lpg2928–lpg2922* locus during intracellular replication of *L. pneumophila*, we examined potential functions of the predicted proteins. Most had putative functional annotations (Fig. 4A). We focused on *lpg2925–lpg2922*, which likely perform a shared function as they are predicted to encode components of an ATP-binding cassette (ABC) transporter. Because of their importance to intracellular replication, we named the genes *lit*, for *Legionella* Infectivity Transporter. Lpg2922 is annotated as an inner-membrane permease (LitI), Lpg2923 as a cytoplasmic ATPase (LitA), Lpg2924 as a periplasmic adaptor protein (LitP), and Lpg2925 as an outer-membrane channel (LitO).

To examine homologs of the predicted transporter, we analyzed the Lit protein sequences by HHpred to find proteins with predicted structural similarity (48–50). LitA contains canonical ATPase motifs, and all but three of the top 100 HHpred matches, each with E-values less than 1e−20, were transporter-associated ATPases. The majority were from Gram-negative and -positive bacteria, but some fungal and human transporter components were among the homologs. The LitI protein is structurally homologous to the MacB ATP-binding permeases from *Acinetobacter baumannii*, *Escherichia coli*, and *Aggregatibacter actinomycetemcomitans*, and a non-canonical ABC transporter permease from *Streptococcus pneumoniae* (51–54) (Table 1). These homologs are inner-membrane proteins that either contain a cytoplasmic ATPase domain or interact with a separate ATPase protein. Proteins homologous to LitP included periplasmic adaptors MacA and AcrA from *E. coli*, a membrane fusion protein from *Pseudomonas aeruginosa*, and heavy metal efflux protein ZneB from *Cupriavidus metallidurans* (52, 55–57) (Table 1). Each of these proteins oligomerizes to bridge together inner- and outer-membrane components of an efflux pump. Finally, LitO was similar to outer-membrane β-barrel proteins from other Gram-negative species, including CmeC from *Campylobacter jejuni*, OprJ from *P. aeruginosa*, and TolC from *E. coli* (52, 58–60) (Table 1). These proteins each associate with additional efflux machinery and function as channels through which substrates are released to the extracellular space. Overall, these results indicate that the LitOPAI proteins exhibit strong similarity to bacterial efflux pump components.

**TABLE 1:** Summary of top protein matches (hits) from HHpred structural homology analysis of transporter components LitI, LitP, and LitO^*a*^

Annotation/description	Species	PDB ID	Expect (*E*) value	Aligned aa	Hit length (aa)
LitI/Lpg2922 (415 aa)					
Macrolide export ATP-binding/permease MacB	*Escherichia coli*	5NIK_K	9.00E−38	399	654
Macrolide export ATP-binding/permease MacB	*Acinetobacter baumannii*	5GKO	1.10E−37	396	671
Non-canonical ABC transporter	*Streptococcus pneumoniae*	5XU1_S	2.30E−37	392	419
Macrolide export ATP-binding/permease MacB	*Aggregatibacter actinomycetemcomitans*	5LJ7_A	1.90E−35	394	664
LitP/Lpg2924 (381 aa)					
Periplasmic membrane fusion protein MacA	*Escherichia coli*	3FPP	2.00E−36	318	341
Multidrug efflux pump subunit AcrA	*Escherichia coli*	5NG5	2.20E−35	311	373
Probable RND efflux membrane fusion protein	*Pseudomonas aeruginosa*	6VEJ	8.40E−35	316	695
ZneB heavy metal efflux protein	*Cupriavidus metallidurans*	3LNN	2.90E−34	322	359
LitO/Lpg2925 (542 aa)					
OM channel CmeC	*Campylobacter jejuni*	4MT4	5.40E−42	415	479
Cation efflux system protein CusC	*Escherichia coli*	4K7R	5.90E−41	443	446
OM protein OprJ	*Pseudomonas aeruginosa*	5AZS_C	7.20E−41	450	468
OM channel MtrE	*Neisseria gonorrhoeae*	4MT0	6.30E−41	441	447
OM protein TolC	*Escherichia coli*	1EK9	7.80E−37	407	428

^*a*^ Query sequence length is indicated next to the protein name. “Aligned aa” indicates the number of query residues that were aligned with residues in the matched protein. aa, amino acids; PDB, Protein Data Bank; OM, outer membrane.

### The LitOPAI transporter exhibits efflux activity but does not alter *L. pneumophila* sensitivity to antimicrobial substrates of related efflux pumps

**Fig 6:**
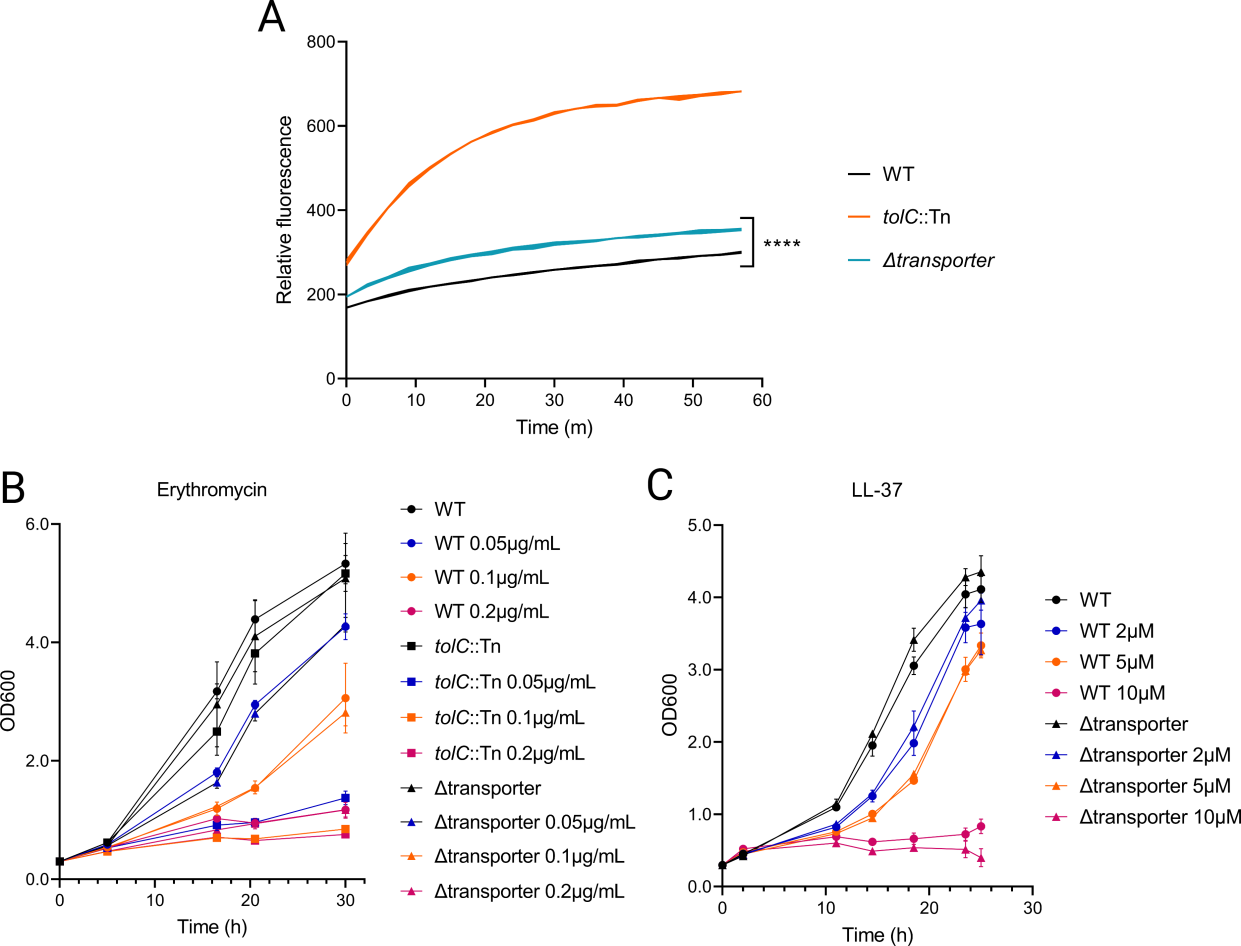
The Lit transporter is capable of reducing ethidium bromide accumulation but does not alter *L. pneumophila* sensitivity to substrates of homologous efflux pumps. (**A**) Relative fluorescence of *L. pneumophila* strains exposed to ethidium bromide. Bacteria were grown on CYE agar, collected, and washed with sterile water. Samples were diluted in water and distributed in black 96-well plates. Fluorescence was monitored starting immediately after addition of ethidium bromide. Line width represents the standard deviation of the mean of three replicate wells. Asterisks indicate statistical significance by one-way ANOVA relative to WT (****P < 0.0001). Results are representative of three independent experiments. (**B and C**) Growth of WT *L. pneumophila*, a *tolC*::Tn mutant, and a Lit transporter-null mutant in liquid media (AYE) containing varied concentrations of erythromycin (**B**) or antimicrobial peptide LL-37 (**C**).

To determine whether the Lit transporter functions as an efflux pump, we measured the fluorescence of *L. pneumophila* exposed to ethidium bromide (EtBr), a commonly used proxy for efflux activity (21, 61–63). Many bacterial efflux pumps can extrude EtBr, preventing its intracellular accumulation. Thus, increased fluorescence signal resulting from intracellular EtBr indicates a diminished capacity for efflux. WT *L. pneumophila* maintained low fluorescence throughout the assay, indicating an ability to prevent EtBr accumulation (Fig. 6A). In contrast, a strain deficient in TolC, an outer-membrane protein associated with multiple exporters, rapidly increased in fluorescence, as expected (21). Mutants lacking the Lit transporter exhibited an intermediate phenotype, accumulating slightly but significantly greater fluorescence than WT bacteria (Fig. 6A). We reasoned that this result was consistent with a strain lacking the function of a single efflux pump, unlike the TolC-deficient mutant, and concluded that LitOPAI exhibits efflux activity.

Because the Lit transporter is homologous to the MacAB-TolC efflux pump, we investigated the relationship between these transporters more closely. The MacAB-TolC pump, comprised of inner- and outer-membrane complexes (MacB and TolC) bridged by a periplasmic adaptor (MacA), was named for its ability to confer macrolide resistance (64). There are no MacAB homologs annotated in the *L. pneumophila* genome. Performing a BLASTp search of the *E. coli* MacB sequence against *L. pneumophila* Philadelphia-1 yields LitA (*E*-value 1e−64) and LitI (*E*-value 5e−43) in the top three matches (65). Repeating this with MacA yields LitP as only the eighth highest match (*E*-value 3e−8). To determine whether LitOPAI functions similarly to MacAB, we compared the growth of WT and a transporter mutant in media containing varied concentrations of the macrolide erythromycin, but observed no difference in sensitivity (Fig. 6B). A mutant deficient in TolC, which influences *L. pneumophila* sensitivity to erythromycin (21), was inhibited at all concentrations tested.

The Spr0693–Spr0694–Spr0695 pump in *S. pneumoniae*, also homologous to LitPAI and MacAB, confers resistance to the antimicrobial peptide LL-37 (54). We tested whether the Lit transporter affected LL-37 sensitivity in *L. pneumophila*, but found no difference between WT and *lit*-deficient cultures in the presence of LL-37 (Fig. 6C). In all, we concluded that the Lit transporter does not accept these substrates of structurally similar efflux pumps. The Lit transporter presumably supports *L. pneumophila* fitness during infection of mammalian cells by exporting a substrate or substrates to the lumen of the LCV, but the nature of the substrate(s) remains unknown.

### Lpg2928–Lpg2926 are predicted nucleotide-modifying enzymes with undefined relationships to LitOPAI function

Lpg2928 is annotated as KsgA, a dimethyladenosine transferase that modifies adenosine residues in 16S rRNA (66). Lpg2928 and *E. coli* KsgA are 49.8% identical and 64% similar at the sequence level (*E*-value 6e−89). The hypothetical protein Lpg2927 is predicted by InterProScan to contain poorly-characterized domains that may confer helicase, relaxase, and/or nickase activity (67, 68). HHpred returned only one match with structural homology throughout the majority of Lpg2927: a protein of unknown function from the closely related pathogen *Coxiella burnetii* (Cbu0560, PDB ID: 3KQ5; *E*-value 1.7e−47) (48–50). Lpg2926 is a predicted homolog of ApaH, a diadenosine tetraphosphatase that hydrolyzes diadenosine tetraphosphate (Ap_4_A) to generate two ADP molecules (69). Alignment of the Lpg2926 sequence with that of *E. coli* ApaH shows 50.2% identity and 63% similarity between the two (*E*-value 2e−89) (65).

The annotations of Lpg2928–Lpg2926 did not suggest an obvious connection to LitOPAI function. To determine whether these proteins function with the Lit transporter, we generated mutants lacking both the *lit* genes and genes upstream. Strains lacking *litPAI* or *litOPAI* replicated similarly to the Δ*litI* mutant in BMDMs, reinforcing that the Lit proteins perform a shared function (Fig. S8A). As expected, a Δ*lpg2926–lpg2922* mutant phenocopied the Δ*litPAI* and Δ*litOPAI* strains because Lpg2926 is dispensable for *L. pneumophila* replication in BMDMs (Fig. 5A). Strains lacking *lpg2927–lpg2922* or *lpg2928–lpg2922* were more severely defective for intracellular replication compared to strains lacking the transporter genes alone, although the difference was not statistically significant (Fig. S8A).

The predicted enzymatic functions of Lpg2928–Lpg2926 share a common theme of nucleotide modification. To determine whether any of these proteins transcriptionally regulate the *lit* genes, we examined gene expression in Δ*lpg2928*, Δ*lpg2927*, and Δ*lpg2926* mutants using qRT-PCR. We found no difference in *litP* expression in any strain compared to WT *L. pneumophila* (Fig. S8B).

## DISCUSSION

We present an examination of determinants influencing *L. pneumophila* fitness during infection of mammalian hosts. Using InSeq technology combined with Tn mutant sublibraries, we identified *L. pneumophila* genes required for survival and replication in A/J mice and BMDMs. Additionally, we identified mutants with intrinsic growth defects unrelated to the intracellular environment. The screens yielded expected results based on previous studies, including the importance of Type IV secretion, Type II secretion, and three Type IV effectors during infection, but also uncovered genes with previously unrecognized contributions to *L. pneumophila* fitness in mammalian hosts.

A comparison of genes supporting bacterial fitness in the three screens showed that the largest overlapping subset was comprised of the genes influencing fitness in both the mouse and in BMDMs. However, larger subsets of genes were identified in only the BMDM screen or the mouse screen. Genes identified only in mice may represent functions promoting bacterial survival in the complex multicellular environment of the lung, where factors produced by cells other than BMDMs work to clear microbes from this privileged niche. Many genes identified only in BMDMs fell just short of significance thresholds in the mouse screen, which may reflect that screening *L. pneumophila* libraries in BMDMs is more sensitive for identifying intracellular fitness determinants compared to screening in mice. This is likely because innate immune responses triggered by *L. pneumophila* in the lungs begin restricting replication in alveolar macrophages early in infection, limiting bacterial expansion more efficiently than BMDMs *ex vivo* (70). Additionally, although BMDMs offer a convenient and robust model for infection of primary cells, physiological differences from alveolar macrophages may affect bacterial requirements for infection (71, 72).

From the genes that significantly contributed to *L. pneumophila* fitness in both BMDMs and mice, we identified 30 that, to our knowledge, have not been previously reported in these infection models. Of these, mutants in *lpg0870*, *lpg2863*, *lpg2341*, and *lpg1728* displayed the most striking phenotypes in both screens.

Multiple mutants in *lpg1728* displayed severe growth defects during infection, with over 1,000-fold lower relative abundance in output populations compared to inputs, indicating a clear requirement for *lpg1728* in these models. In a previous study, disruption of *lpg1728* resulted in diminished *L. pneumophila* replication in the protozoan host *Acanthamoeba polyphaga*, slight replication defects in U-937 cells, and deficiencies in host-cell killing in both models (73). The function of this predicted inner-membrane protein, named *pmiA* (protozoan and macrophage infectivity A), remains unknown.

Lpg0870 is annotated as enoyl-CoA hydratase, an enzyme in the β-oxidation pathway of fatty acid metabolism. β-Oxidation may or may not be linked to poly-3-hydroxybutyrate (PHB) synthesis via production of acetyl-CoA, based on conflicting isotopolog profiling results (74, 75). PHB is synthesized by *L. pneumophila* during late- and post-exponential growth, and catabolized during stationary phase (74). Since PHB is an important carbon and energy source during starvation, it may be crucial to the infection cycle (75).

Lpg2863, annotated as pteridine reductase 1, was shown to be required for optimal infection of *A. polyphaga* and *Vermamoeba vermiformis* (28). Pterins are highly-conserved enzymatic cofactors required for all known phenylalanine hydroxylases in proteobacteria (76). Phenylalanine hydroxylase performs the first step in L-Phe breakdown; in *L. pneumophila*, this pathway has been connected to production of pyomelanin, a pigment participating in iron assimilation (77, 78).

Finally, Lpg2341 is a predicted member of the DnaJ family and a homolog of *E. coli* HSP40, a well-studied cochaperone of HSP70 that prevents protein misfolding and aggregation (79, 80). The strong defect we observed is unsurprising, as mutants in *lpg2341* showed significant fitness defects in four amoebal species and in U-937 cells (28).

We were intrigued that Tn mutants in multiple genes within the *lpg2928–lpg2922* cluster displayed robust fitness defects in both the BMDM and mouse screens. Homology predictions indicate that the proteins encoded by *lpg2925–lpg2922*, now designated *litOPAI*, are components of an ABC transporter. Lpg2928 is homologous to KsgA, which methylates adenosine residues in rRNA (66). The significance of these modifications is unknown, as KsgA is dispensable for growth in *E. coli* (66). The hypothetical protein Lpg2927, which has a homolog in *C. burnetii*, may have enzymatic functions related to DNA modification. Lpg2926 is annotated as ApaH, which hydrolyzes the nucleotide Ap_4_A. There is evidence that Ap_4_A, which is produced by prokaryotes and eukaryotes, may act as a secondary messenger to actively facilitate stress responses (69). We observed that an in-frame *lpg2926* deletion mutant had no detectable replication defect in BMDMs, unlike the *lpg2926*::Tn mutant. A previous study found that *lpg2926*::Tn mutants were attenuated in *Acanthamoeba castellanii* (28); however, this may have also resulted from disruption of downstream gene expression. Loss of *lpg2928* and *lpg2927* in addition to *litOPAI* resulted in a slightly enhanced replication defect in BMDMs, and Lpg2928–Lpg2926 do not influence *lit* gene expression at the transcriptional level. Overall, it remains unclear whether the activities of Lpg2928, Lpg2927, and Lpg2926 are linked to the LitOPAI transporter.

The LitOPAI transporter is homologous to an unusual category of ABC transporters known as the Type VII superfamily. The archetypal member of this family, MacAB-TolC, spans both bacterial membranes but accepts substrates from the periplasm for translocation to the extracellular space (52, 81–83). The eight transmembrane helices of dimerized MacB are fewer than any other known transporter fold (81). Alignment of LitI with *E. coli* K-12 MacB shows 29% identity and 49% similarity between the sequences (*E*-value 1e−44) (65). A ColabFold-generated model of LitI aligns well with the MacB structure, particularly in the transmembrane domains (84–87) (Fig. S9A). A key difference between the two is that MacB contains both transmembrane and ATPase domains, while the ATPase LitA is encoded separately from LitI. Models of LitI and LitA align together to the structure of MacB (Fig. S9B and C). Encoding permease and ATPase functions in separate proteins is more typical of ABC importers (88, 89). Thus, while LitOPAI shares many features with MacAB-TolC, it does not fit precisely into the known families of ABC transporters. Interestingly, the Spr0693–Spr0694–Spr0695 and YknWXYZ transporters of Gram-positive bacteria *S. pneumoniae* and *Bacillus subtilis*, respectively, are also exporters with distinct ATPase and permease proteins (54, 90). However, LitOPAI does not appear to share the ability of Spr0693–Spr0694–Spr0695 to efflux LL-37.

We similarly used ColabFold to model LitP, the periplasmic bridge between LitI and LitO. LitP has a similar domain architecture to MacA, which contains an α-helical hairpin domain, contacting the outer-membrane channel; a lipoyl domain, contacting other MacA monomers to form a hexameric ring; and the β-barrel and membrane-proximal domains, contacting the periplasmic domain of the permease (52). The LitP model aligns slightly less well with MacA than LitI with MacB structurally (Fig. S10) and by sequence alignment (20% identity, 43% similarity, *E*-value 1e−11). Alignment of the LitP and MacA structures revealed that both α-helices forming the LitP hairpin domain are shorter than in MacA, and two small helices protrude from this domain in LitP that are non-existent in MacA (Fig. S10). Because LitOPAI likely accepts substrates from the periplasm, these disparities, as well as structural differences in the globular domains of the permeases, may be connected to differences in substrate specificity (52, 83).

**Fig 7:**
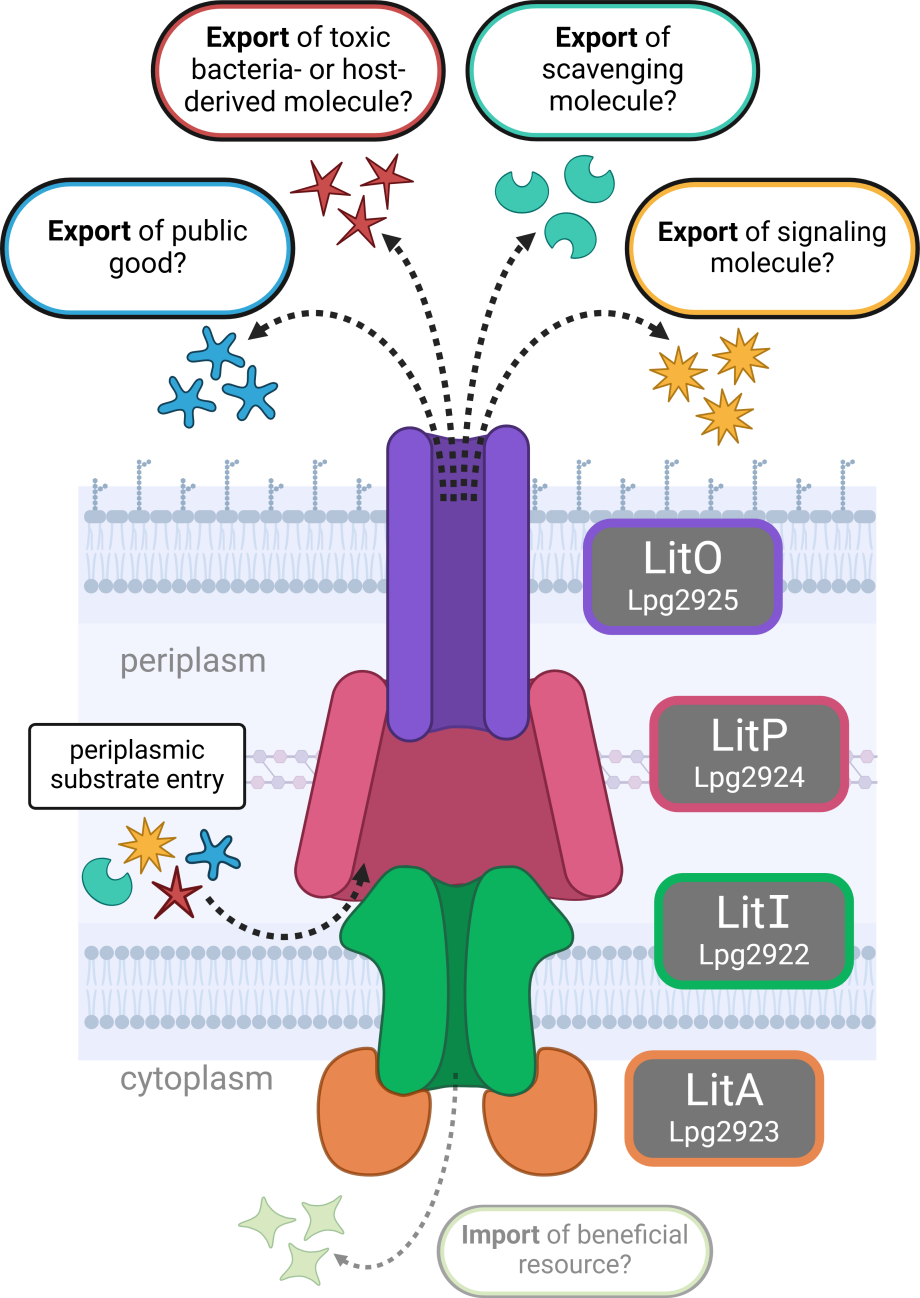
Model of predicted subcellular localization of LitOPAI (Lpg2925-Lpg2922) proteins and putative transporter functions during intracellular replication of *L. pneumophila*. Depicted are the locations of the transporter proteins in the bacterial cell wall based on homology to other efflux pumps. Possible functions of this transporter include the export of toxic molecules of bacterial or host origin, export of a scavenging molecule, secretion of a signaling molecule, or release of a public good such as a host-modulating toxin or extracellular detoxification mechanism. Import of a molecule beneficial to intracellular fitness is unlikely but cannot currently be ruled out. Created with BioRender.com.

Through a genetic screening approach using low-complexity Tn libraries, we identified an ABC transporter that contributes to *L. pneumophila* fitness in both animal and primary macrophage infection models. Many open questions remain about the role of LitOPAI in the host–pathogen interaction, but the most intriguing regards the nature of its physiologically relevant substrate(s). Although we cannot currently rule out that LitOPAI may import a nutrient that supports replication, it more likely functions as an exporter due to its extensive similarity to known efflux pumps. Multidrug efflux pumps transport diverse molecules, making it a challenge to identify native substrates. We are considering several hypotheses about the types of molecules *L. pneumophila* would benefit from transporting to the vacuole lumen (Fig. 7). One possibility is that the substrate is a toxic molecule that must be expelled from the cell, such as a metabolic byproduct or a host-derived molecule like an antimicrobial peptide (54). Another possibility is that the transporter releases a signal for interbacterial communication in the vacuole, similar to a quorum sensing mechanism. There is evidence that proton-driven pumps in *P. aeruginosa* and *E. coli* are regulated by quorum sensing and can secrete non-diffusible autoinducers (91, 92). Also in *P. aeruginosa*, the siderophore pyoverdine, important for both iron scavenging and regulation of virulence factors via surface signaling, is secreted by a MacAB-TolC homolog called PvdRT-OpmQ (93, 94). Thus, another potential role for LitOPAI is the release of a molecule that scavenges a key nutrient. Finally, the transporter may secrete a public good, such as a host-modulating toxin or an extracellular detoxification mechanism. Both of these substrate functions have been demonstrated for MacAB-TolC; in *E. coli*, heat-stable enterotoxin II is delivered to the host via MacAB-TolC (83), while in *Salmonella enterica* serovar Typhimurium, modified siderophore products neutralize extracellular reactive oxygen species in a MacAB-TolC-dependent manner (95, 96). Continued studies are focused on refining our understanding of the specific role of LitOPAI in promoting *L. pneumophila* intravacuolar replication.

## MATERIALS AND METHODS

### Bacterial strains, plasmids, and culture conditions

Cloning was performed in *E. coli* Mach1 (expression plasmids; Invitrogen) or DH5α-*λpir* (deletion constructs [31, 97]). *E. coli* were cultured at 37°C in lysogeny broth (LB [98]; glucose omitted) (Mach1) or 2× yeast extract–tryptone medium (99) (DH5α), with 50 µg/mL of kanamycin or 25 µg/mL of chloramphenicol where appropriate. *L. pneumophila* strains were derived from SRS43 (31), wherein the thymidine auxotrophy of laboratory strain Lp02 was reversed. *L. pneumophila* were maintained at 37°C in supplemented *N-*(2-acetamido)-2-aminoethanesulfonic acid (ACES)-buffered yeast extract (AYE) or charcoal ACES-buffered yeast extract agar (CYE) as described (100, 101), with 100 µg/mL of streptomycin. As appropriate, media contained 5 µg/mL of chloramphenicol (Tn mutants), 10 µg/mL of chloramphenicol (plasmid maintenance), 15 µg/mL of kanamycin (allelic exchange), or 25 µg/mL of kanamycin (plasmid maintenance). For experiments with individual Tn mutants, single colonies were isolated from the arrayed Tn library (31), and the genomic locations of the Tn insertions were verified using PCR. For strains containing inducible plasmids, 0.5 mM isopropyl β-D-1-thiogalactopyranoside (IPTG) was added. Plasmids from this study are listed in Table 2.

**TABLE 2:** Plasmids used in this study

Name	Description	Source
pSR47s	Vector backbone for allelic exchange constructs	(102, 103)
pSR47s::Δ*lpg2922*	Deletion of *lpg2922*	This study
pSR47s::Δ*lpg2923*	Deletion of *lpg2923*	This study
pSR47s::Δ*lpg2924*	Deletion of *lpg2924*	This study
pSR47s::Δ*lpg2925*	Deletion of *lpg2925*	This study
pSR47s::Δ*lpg2926*	Deletion of *lpg2926*	This study
pSR47s::Δ*lpg2927*	Deletion of *lpg2927*	This study
pSR47s::Δ*lpg2928*	Deletion of *lpg2928*	This study
pJB1806 (pEV)	*L. pneumophila* expression vector	(104)
pJB1806::*lpg2922*	IPTG-inducible expression of *lpg2922*	This study
pJB1806::*lpg2923*	IPTG-inducible expression of *lpg2923*	This study
pJB1806::*lpg2924*	IPTG-inducible expression of *lpg2924*	This study
pJB1806::*lpg2925*	IPTG-inducible expression of *lpg2925*	This study
pJB1806::*lpg2927*	IPTG-inducible expression of *lpg2927*	This study
pJB1806::*lpg2928*	IPTG-inducible expression of *lpg2928*	This study

### Mice and BMDMs

Female, 7- to 10-week-old A/J mice (Jackson Labs 000646) were used in intranasal infection experiments. A/J BMDMs were differentiated as described (105) in Roswell Park Memorial Institute media (RPMI) with 20% heat-inactivated fetal bovine serum (FBS) and 15% L929-conditioned supernatant, and maintained at 37°C, 5% CO_2_.

### Sublibrary generation

An arrayed library of 10,163 *L*. *pneumophila* Tn mutants (31), stored in 96-well plates, was thawed on ice. Eight microliters of each mutant was spotted onto solid media, so each plate contained the mutants from one 96-well dish (Fig. S1B). After 4 days of growth, pools were generated by collecting bacteria from each plate in water. Pools were diluted to equal optical density (OD600) and combined in equal volumes to make 15 sublibraries, each containing 500–700 mutants. Sublibraries were combined with 2× freezing medium (4% peptone, 10% glycerol) and stored in single-use aliquots at −70°C.

### BMDM and mouse screens

For each experiment, a sublibrary aliquot was thawed and incubated for 3 days on CYE to generate a lawn of bacteria. Bacteria were collected and used to start AYE cultures that were grown to OD600 3.3–3.9. Culture samples were collected as input populations in technical replicates. BMDMs were seeded in 12-well plates at 5 × 10^5^ cells/well approximately 24 h pre-infection. The inoculum was prepared in replating media (RPMI, 10% FBS, 7.5% L929-conditioned supernatant) to infect at MOI 0.1 as described (31). Plates were centrifuged at 200 × *g* for 5 min to synchronize infection. At 48 h post-infection, supernatants were collected, and BMDMs were hypotonically lysed in water. Lysates were combined with supernatants, vortexed, and plated on 15-cm CYE plates to generate output populations (31). Serial dilutions were plated to enumerate CFUs in the inoculum and 48 h post-infection. Each sublibrary was tested in 10 wells of BMDMs, split across two independent experiments.

A/J mice were anesthetized with 100 mg/kg of ketamine, 10 mg/kg of xylazine in PBS via intraperitoneal injection and intranasally infected with 5 × 10^5^ bacteria in PBS (31, 106). At 4 h (*n* = 3) and 48 h post-infection (*n* = 9–10), mice were euthanized by CO_2_ asphyxiation, and lungs were harvested and homogenized in sterile water (Bullet Blender, Next Advance). Homogenates were adjusted to a volume of 1 mL in water, and serial dilutions were plated for CFU determination. Remaining homogenate was plated on CYE to generate output populations. All inputs and outputs were collected, pelleted, and stored at −20°C until InSeq library preparation. Outputs for each sublibrary were collected from 9 to 10 mice, split across two independent experiments.

Please refer to the supplemental material for additional methods.

## SUPPLEMENTAL MATERIAL

Data Set S1 mbio.01955-24-s0001.xlsx InSeq data from BMDM screen.

Data Set S2 mbio.01955-24-s0002.xlsx InSeq data from mouse screen.

Data Set S3 mbio.01955-24-s0003.xlsx InSeq data from screen on CYE media.

Data Set S4 mbio.01955-24-s0004.xlsx Summary of InSeq screens.

Data Set S5 mbio.01955-24-s0005.xlsx Overlapping hits from InSeq screens.

Supplemental material mbio.01955-24-s0006.pdf Additional methods, tables, and figures.

ASM does not own the copyrights to Supplemental Material that may be linked to, or accessed through, an article. The authors have granted ASM a non-exclusive, world-wide license to publish the Supplemental Material files. Please contact the corresponding author directly for reuse.
