# Supplementary material for: InSeq analysis of defined Legionella pneumophila libraries identifies a transporter-encoding gene cluster important for intracellular replication in mammalian hosts
Source: mBio. 2024 Oct 4;15(11):e01955-24. doi: 10.1128/mbio.01955-24 (PMC11559062; doi:10.1128/mbio.01955-24)
Supplement: Supplemental material — Additional methods, tables, and figures. [file mbio.01955-24-s0006.pdf]

InSeq analysis of defined *Legionella pneumophila* libraries identifies a transporter-encoding gene cluster required for optimal infection of mammalian hosts.

Caitlin E. Moss and Craig R. Roy

SUPPLEMENTAL INFORMATION

SUPPLEMENTAL MATERIALS AND METHODS

Media Screen

For each experiment, a sublibrary aliquot was thawed on ice, plated on CYE, and incubated for 3 days. The resulting bacteria were collected and diluted to OD600 0.1. Samples of this were pelleted and stored at -20°C for preparation of input libraries in two technical replicates. 100μL of diluted bacteria were plated on each 15cm plate (n=8 per sublibrary), incubated for 3 days at 37°C, collected in sterile water, pelleted, and stored for preparation of output libraries.

Production of InSeq libraries

InSeq libraries were generated as described (1-3). Each library was marked with a unique 6-base pair barcode within the sequencing adapter. Purified, adapter-ligated PCR products for each library were adjusted to 10nM, combined in equal volumes, and submitted to the Yale Center for Genome Analysis for Illumina HiSeq 2500 sequencing.

Statistical analysis, data filtration, and functional analysis

InSeq analysis was performed as described (1-3). Input and output library sequencing reads were normalized to read counts per million (CPM). Within each sublibrary, data from multiple mutants with insertions in the same gene were consolidated to obtain input and output counts by gene. To eliminate 0-values for subsequent analyses, a value of 1 was added to all read counts. CPM values were used to generate $\log_{10}$ output:input ratios for each gene. A z-test followed by False Discovery Rate correction were performed to evaluate statistical significance for each sublibrary ($q<0.05$) as previously described (2, 3). Entries with input values of fewer than 5 CPM were removed from further analysis. Z-scores were calculated for each gene by subtracting the population mean $\log_{10}$ ratio from the $\log_{10}$ ratio for each gene, then dividing by the population standard deviation. Results were filtered to identify genes from each sublibrary with $q<0.05$ and $Z>1$ or $Z<-1$, yielding lists of significant hits in each of the 15 sublibraries for each of the three screens. To create one comprehensive list of significant hits for each screen, the results from individual sublibraries were consolidated by determining the frequency at which a gene met the significance criteria ($q<0.05$; $Z>1$ or $Z<-1$) when it was tested via independent mutants in multiple sublibraries. Genes for which mutants met the thresholds in >60% of the sublibraries in which they were tested were used to generate final lists of hits for each screen. In the rare case where individual mutants in a gene showed a mix of positive and negative Z-scores in the same screen, the gene was excluded from further analysis. Intergenic mutants were also excluded.

Bioinformatic Analyses

Clusters of Orthologous Genes (COG) analysis was performed using the 2020 update of the NCBI COG definitions (4, 5). The HHpred tool from the MPI Bioinformatics Toolkit was used to search for structurally homologous genes based on amino acid sequence, with default parameters (6-8). Amino acid sequence alignments were performed using NCBI BLASTp with default parameters (9). Protein modeling was performed with ColabFold v1.5.5, based on AlphaFold2 and MMseqs2, using default parameters, and first ranked prediction was used for structural alignments (10-13). Structures were visualized and aligned using the PyMOL Molecular Graphics System, v2.6.0a0 (14).

BMDM Growth Curves

Differentiated A/J BMDMs were seeded in 24-well plates at $2.5x10^5$ cells/well in replating medium. Approximately 24h later, cells were infected (MOI 0.1) using bacteria patched from a single colony, grown for 48h, collected, and diluted in replating media. Following inoculation, plates were centrifuged at 200g for 5min at room temperature before incubation to synchronize the infection. A sample of the inoculum was diluted and plated for CFU enumeration. At 24h, 48h, and 72h post-infection, supernatants were collected and combined with BMDM hypotonic lysates. Serial dilutions were plated for CFU enumeration at each timepoint.

Mouse infections

Overnight AYE cultures were started from 48h patches and grown to OD600 3.2-3.9. Inocula were prepared and mice were anesthetized with ketamine/xylazine and intranasally infected

with $5x10^5$ bacteria. (n≥3 per bacterial strain). At 4h and 48h post-infection, mice were sacrificed and lungs were homogenized, diluted, and plated for CFU enumeration.

AYE Growth Curves

AYE cultures were inoculated from 48h patches at OD600 0.3 (n=3 per strain). The OD600 was measured periodically until cultures began to enter post-exponential phase. Erythromycin (Sigma) or LL-37 (human; MedChemExpress) were added at the noted concentrations, as appropriate.

Plasmid construction and deletion strain generation

Plasmids were constructed using sequence- and ligation-independent cloning (SLIC). Clones were screened for the plasmid insert by PCR, and subsequently confirmed via Sanger sequencing (Keck DNA Sequencing Facility, Yale University). Plasmids were transformed into *L. pneumophila* via electroporation (Bio-Rad GenePulser Xcell). Chromosomal deletions in *L. pneumophila* were generated by allelic exchange using the suicide vector pSR47S as described (15, 16). Sucrose-resistant, kanamycin-sensitive clones were screened by PCR to verify deletion.

Ethidium bromide accumulation

WT, *tolC*::Tn, and Lit-null *L. pneumophila* were collected from two-day patches on CYE agar into water, washed, and the OD600 was measured. Suspensions at OD 0.3 were prepared and distributed into Corning Costar 96-well flat-bottom black plates at 100 µL/well. 3 µL of a 100 µg/mL solution of ethidium bromide (Sigma) in water was added to wells as appropriate, just

before inserting into the plate reader (Tecan Spark). Fluorescence (excitation 518, emission 605) was measured every 3 minutes. Gain was set to 30% based on a *tolC*::Tn-containing well at the beginning of an experiment. Nine measurements were taken per well and averaged. For each experiment, controls lacking ethidium bromide were included in triplicate for each strain, averaged, and subtracted from the corresponding ethidium bromide-containing samples.

Gene expression by qRT-PCR

Bacterial samples from CYE plates were preserved using RNAprotect Bacteria Reagent (Qiagen), and RNA was extracted using the RNeasy Mini kit (Qiagen), with on-column DNase treatment. Following elution, a second DNase digestion was completed in solution and RNA was re-purified using the Zymo Clean & Concentrator kit. RNA was quantified via Nanodrop and equal amounts of RNA from each sample were reverse-transcribed using the iScript Advanced cDNA Synthesis Kit (Bio-Rad) according to manufacturer instructions. qPCRs were performed using iQ SYBR Green Supermix in a CFX Connect Real-Time System (Bio-Rad). Relative expression was calculated with the ΔΔCt method, using *gyrB* as the reference. Primer pairs, manufactured by Integrated DNA Technologies: *gyrB*-F (GCTTTGGATATTGCCGGTTTAC), *gyrB*-R (GCAGAGTCACCCTCAACTAAAT); *litP*-F (CATTTGGCAGGCATCAACTG), *litP*-R (CAGCGGTAAAGGAGGGTAATC).

Data availability

All data sets generated by this study are included in this publication.

SUPPLEMENTAL TABLES

Table S1. Overview of transposon mutant composition for the 15 sublibraries, named A through O.

Sublibrary	Number of mutants expected based on original mapping *	Mutants "missing" #	Percent of mutants accounted for	Predicted missing (poor growth) &
A	704	23	96.7	10
B	608	9	98.5	5
C	681	2	99.7	2
D	646	16	97.5	2
E	707	4	99.4	0
F	701	18	97.4	8
G	733	19	97.4	4
H	661	10	98.5	0
I	670	15	97.8	2
J	714	14	98	1
K	711	5	99.3	3
L	660	5	99.2.	0
M	747	9	98.8	2
N	683	11	98.4	1
O	472	16	96.6	0

*Mutants expected in each sublibrary based on the combinatorial sequencing map from Shames, 2017. #Number of expected mutants that were not detected by sequencing input populations during screening. &Number of "missing" mutants that exhibited very light or no growth on solid media during sublibrary production.

Table S2. Genes identified as important for infection in the BMDM screen, mouse screen, or both, that were previously demonstrated to play a role during infection of mammalian hosts.

Lpg number(s)	Name(s)	BMDM hit? (Yes/No)	Mouse hit? (Yes/No)	Function	Citation(s)
lpg0376	*sdhA*	Y	Y	dot/icm effector; LCV integrity	(17, 18)
lpg0441-lpg0444	*icmTSRQ*	Y	Y	type IV secretion	(19, 20)
lpg0446	*icmO*	N	Y	type IV secretion	(19, 20)
lpg0448-lpg0451	*icmMLKE*	Y	Y	type IV secretion	(19, 20)
lpg0452	*icmG*	Y	N	type IV secretion	(19, 20)
lpg0453-lpg0456	*icmCDJB*	Y	Y	type IV secretion	(19, 20)
lpg0458	*icmF*	Y	Y	type IV secretion	(19, 20)
lpg0459	*icmH*	Y	N	type IV secretion	(19, 20)
lpg0472	*dotV*	Y	Y	type IV secretion	(19, 20)
lpg0525	*lvgA*	Y	Y	dot/icm coupling complex	(21, 22)
lpg0540	*phtA*	Y	Y	threonine transport	(23)
lpg0699	*tolC*	N	Y	outer membrane protein	(24)
lpg1319-lpg1320	*lspED*	Y	N	type II secretion	(25)
lpg1358-lpg1359	*lspKJ*	Y	N	type II secretion	(25)
lpg1362	*lspG*	N	Y	type II secretion	(25)

lpg1363	*lspF*	Y	N	type II secretion	(25)
lpg1524	*pilD*	Y	N	prepilin-like peptidase	(25)
lpg1728	*pmiA*	Y	Y	?	(26)
lpg1874	*lspL*	Y	N	type II secretion	(25)
lpg2505	*mesI*	Y	Y	*dot/icm* metaeffector	(2, 27)
lpg2674-lpg2676	*dotDCB*	Y	Y	type IV secretion	(19, 20)
lpg2686	*dotA*	Y	Y	type IV secretion	(19, 20)
lpg2687-lpg2689	*icmVWX*	Y	Y	type IV secretion	(19, 20)
lpg2815	*mavN*	Y	Y	dot/icm effector; iron acquisition	(28)

SUPPLEMENTAL FIGURES

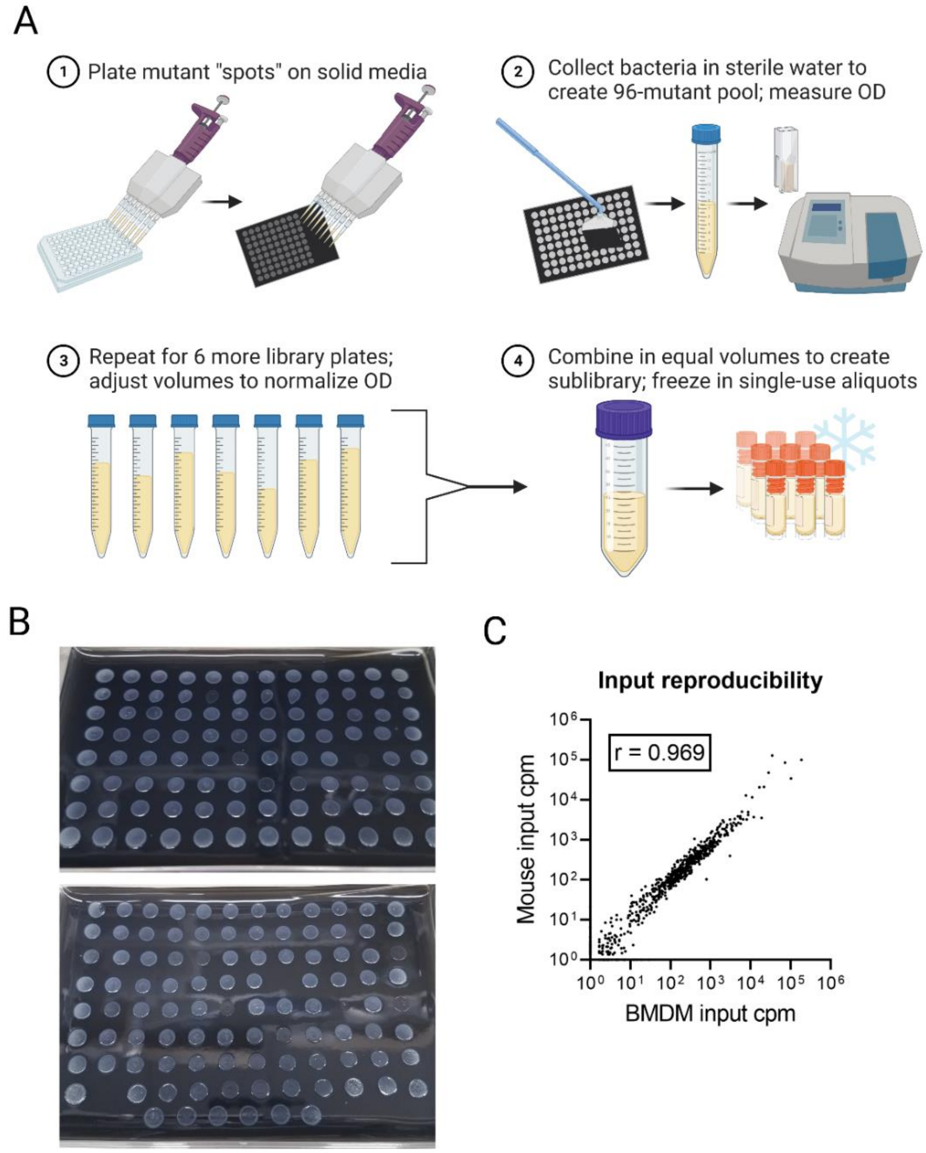

Figure S1. Construction of *L. pneumophila* transposon (Tn) sublibraries for genetic screening. (A) Workflow diagram of sublibrary generation from an arrayed *L. pneumophila* Tn library. Briefly, samples of Tn mutants were grown in “spots” on solid media from 96-well plate stocks. Bacteria on each plate were collected to form a pool. The OD600 of each pool was used to adjust them to the same density. Seven pools were combined in equal volumes to make a sublibrary containing 500-700 mutants and aliquots were stored at -70°C. Created with BioRender.com. **(B)** Photographs of representative Tn mutant “spots” on solid media. **(C)** Reproducibility of sublibrary input populations, as indicated by normalized sequencing read

counts (counts per million, cpm) of Tn mutants cultured from individual single-use aliquots of the same sublibrary for independent experiments on different days. A sublibrary (Sublibrary M) was chosen at random to be displayed here as a representative result. Each point represents the relative abundance of sequencing reads corresponding to a single gene. Spearman's correlation coefficient (r_s) = 0.969.

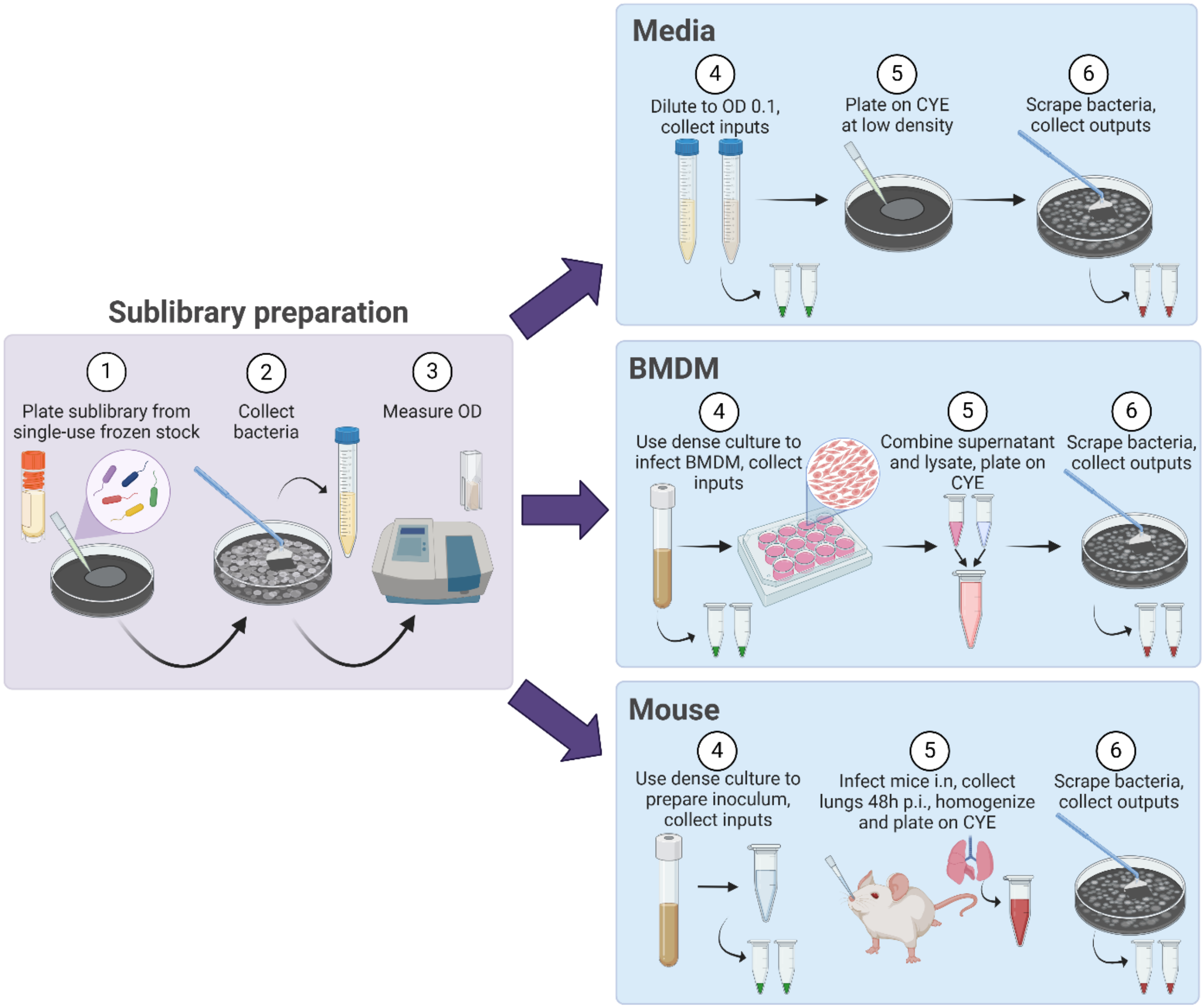

Figure S2. InSeq screening workflow. For each screen, a sublibrary aliquot was thawed and cultured on solid media. Bacteria were suspended in sterile water and the OD600 was measured. For media screens, the suspension was diluted and samples were saved for sequencing library preparation (input samples) or plated on solid media at low density, incubated, and collected (output samples). For BMDM screens, sublibraries were cultured in liquid media (AYE) to an OD600 of 3.3-3.9. Culture samples were preserved for input library preparation. Bacteria were diluted in BMDM re*plating media to inoculate differentiated host cells (MOI 0.1). After 48h, culture supernatants and BMDM lysates were combined and plated on solid media for output samples and CFU enumeration. For mouse screens, AYE cultures and input samples were prepared as above. Bacteria were diluted in sterile PBS to intranasally infect A/J mice. After 48h, lung tissue was harvested, homogenized, and plated on solid media for output samples and CFU enumeration. Created with BioRender.com.

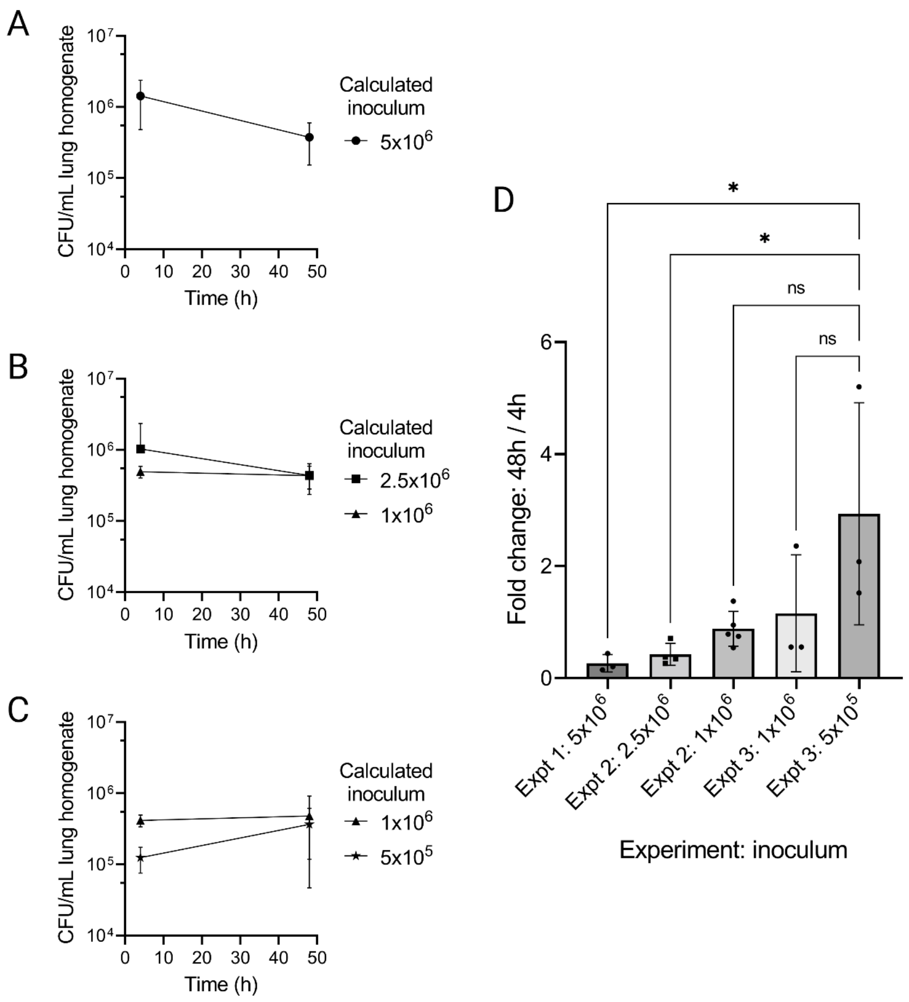

Figure S3. Determination of mouse inoculum for InSeq screening. A/J mice were intranasally infected with wild-type *L. pneumophila* at various inoculum concentrations in three trial experiments, **(A)**, **(B)**, and **(C)**. After 4h (n=2) or 48h (n=3 to 5), lung tissue was harvested, homogenized in sterile water, and plated for CFU enumeration. The data shown in **(A-C)** are summarized in **(D)** as fold change in CFU at 48h post-infection compared to 4h. Asterisks indicate statistical significance evaluated by one-way ANOVA followed by Tukey multiple comparisons test (* $p<0.05$).

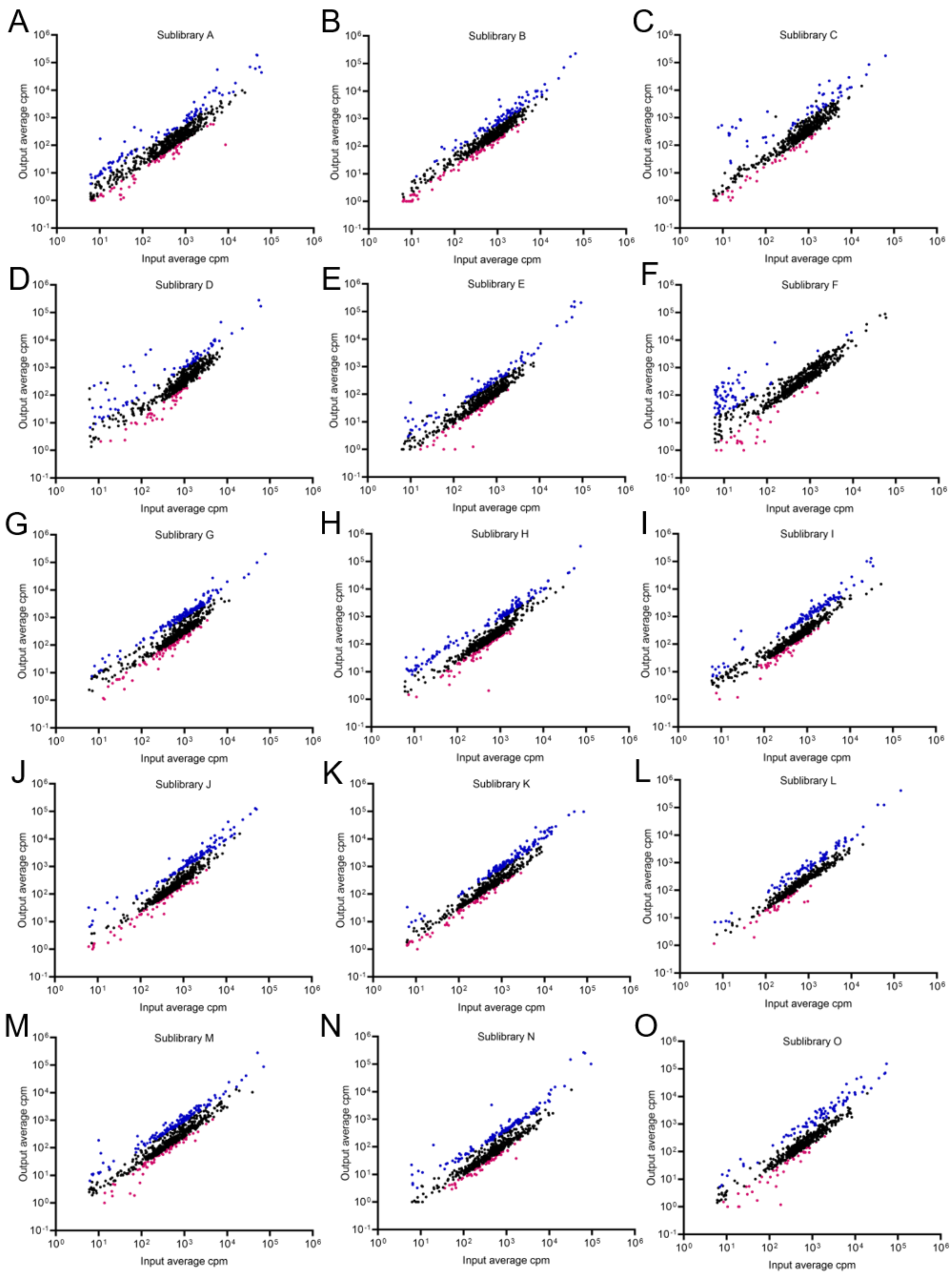

Figure S4. Summary of media InSeq screen results. Average normalized sequencing read counts (counts per million; cpm) of input and output populations from the CYE media screen.

Each point represents the relative abundance of sequencing reads corresponding to Tn mutants in a single gene. **(A-O)** display the data from each mutant sublibrary, A through O. Black dots indicate genes with neutral effects on fitness. Magenta dots indicate genes that support fitness on CYE ($q<0.05$, $Z<-1$; mutants were underrepresented). Blue dots indicate genes that reduce fitness on CYE ($q<0.05$, $Z>1$; mutants were overrepresented).

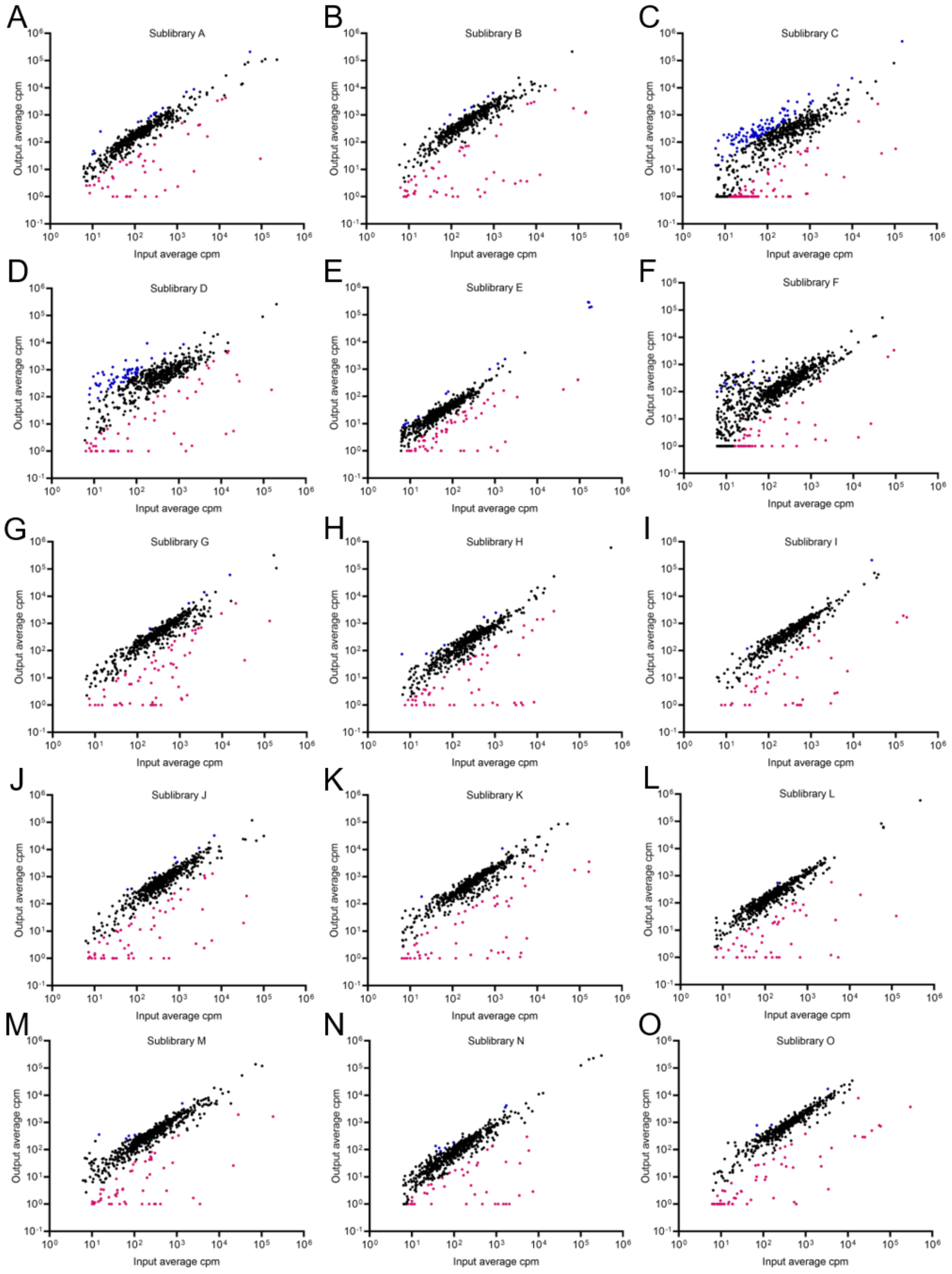

Figure S5. Summary of BMDM InSeq screen results. Average normalized sequencing read counts (counts per million; cpm) of input and output populations from the BMDM screen. Each

point represents the relative abundance of sequencing reads corresponding to Tn mutants in a single gene. **(A-O)** display the data from each mutant sublibrary, A through O. Black dots indicate genes with neutral effects on fitness. Magenta dots indicate genes that support fitness during infection of BMDMs ($q<0.05$, $Z<-1$; mutants were underrepresented). Blue dots indicate genes that reduce fitness in BMDMs ($q<0.05$, $Z>1$; mutants were overrepresented).

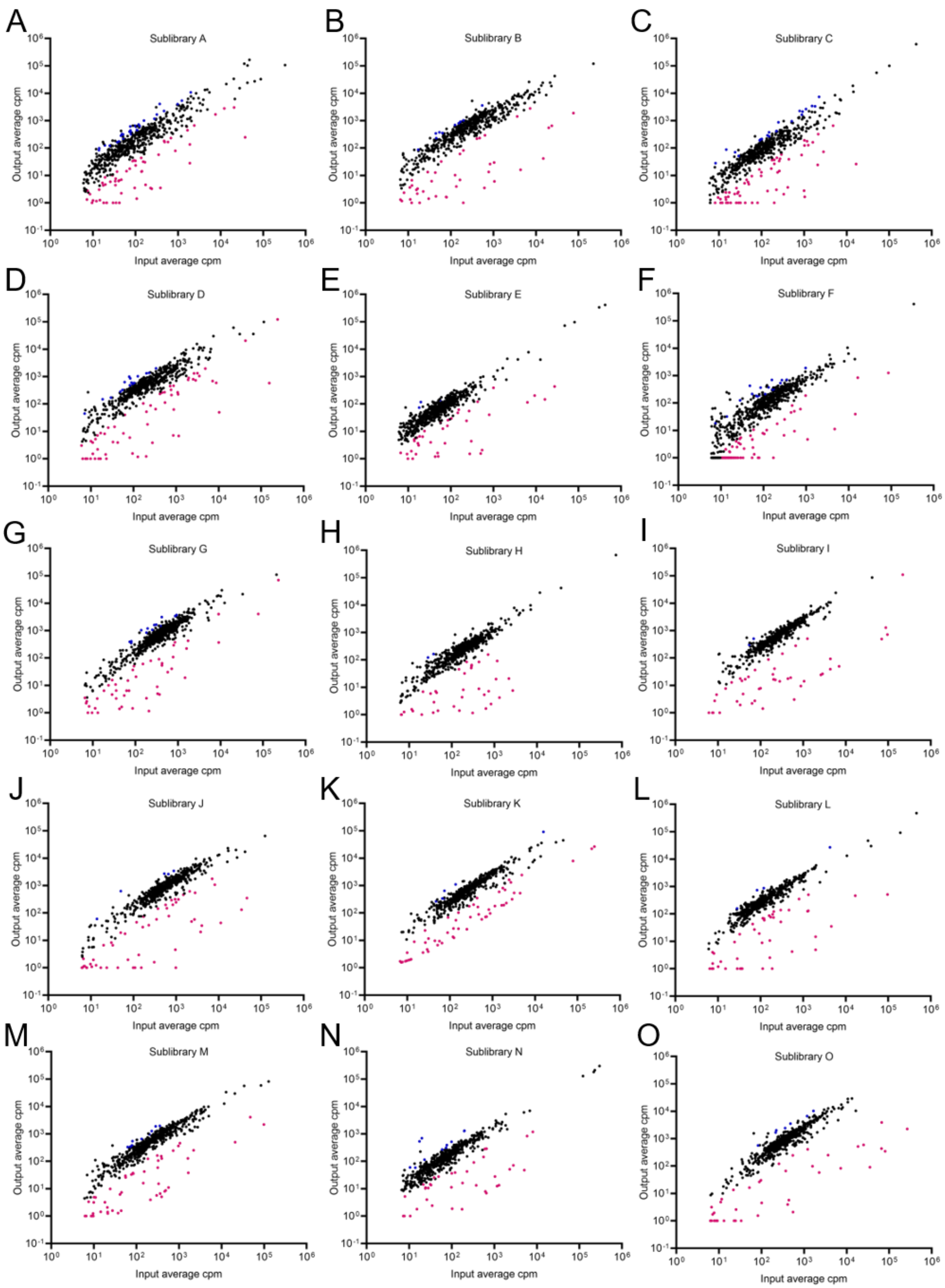

Figure S6. Summary of mouse InSeq screen results. Average normalized sequencing read counts (counts per million; cpm) of input and output populations from the mouse screen. Each

point represents the relative abundance of sequencing reads corresponding to Tn mutants in a single gene. **(A-O)** display the data from each mutant sublibrary, A through O. Black dots indicate genes with neutral effects on fitness. Magenta dots indicate genes that support fitness during infection of mice ($q<0.05$, $Z<-1$; mutants were underrepresented). Blue dots indicate genes that reduce fitness in mice ($q<0.05$, $Z>1$; mutants were overrepresented).

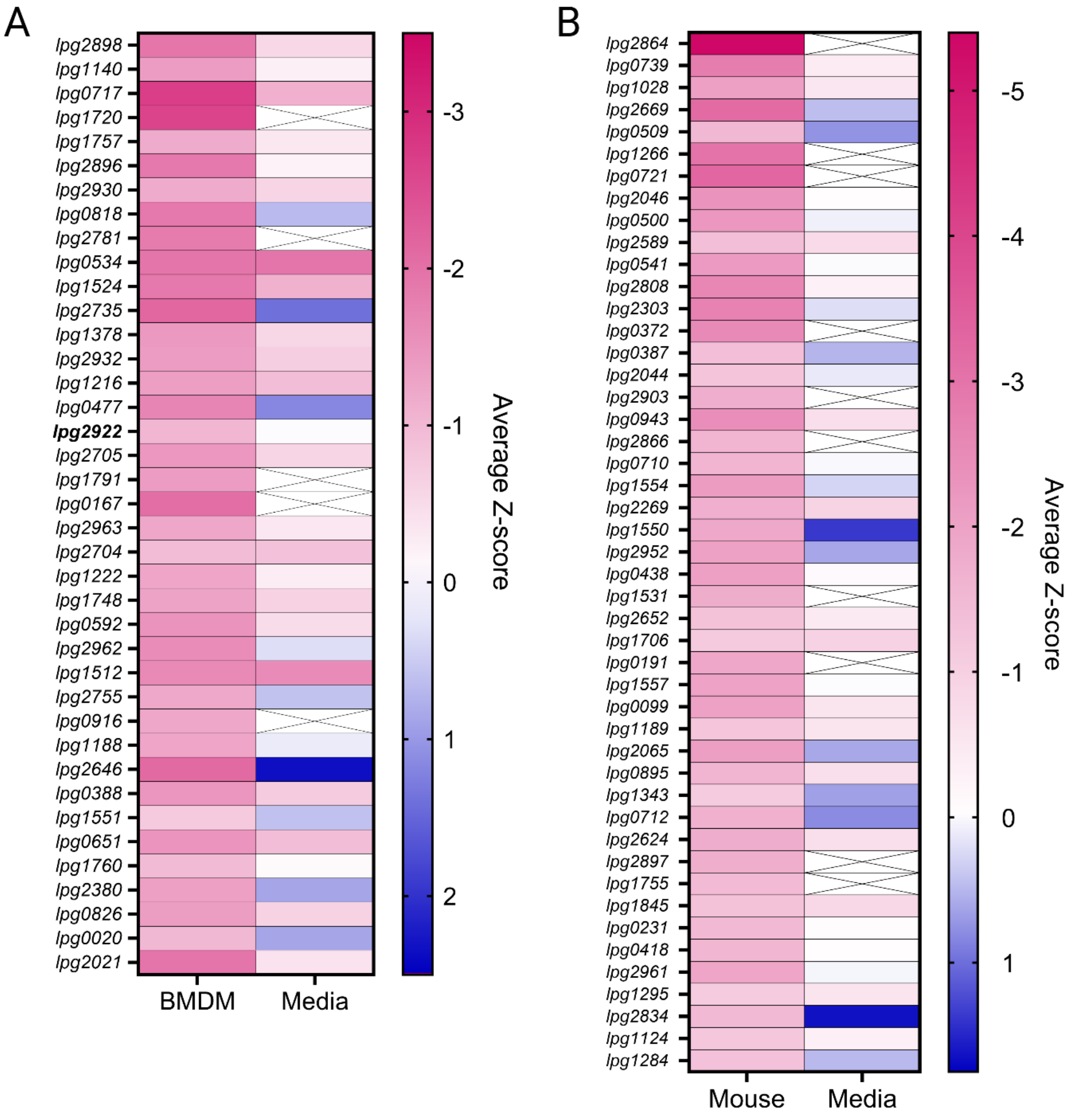

Figure S7. BMDM-only and mouse-only screen hits. Heat maps indicating genes that were significantly underrepresented in the **(A)** BMDM and **(B)** mouse screens. Colors indicate the average Z-score of Tn mutants in a given gene, where magenta specifies mutants that were underrepresented in a screen (genes disruption causes a fitness defect) and blue indicates mutants that were overrepresented (gene disruption confers an advantage). Each column represents results from one screen. The hits are ranked by q-value from the BMDM screen **(A)**, or the mouse screen **(B)**, with the most significant results at the top. Genes from Table S2 have been omitted. X; no data.

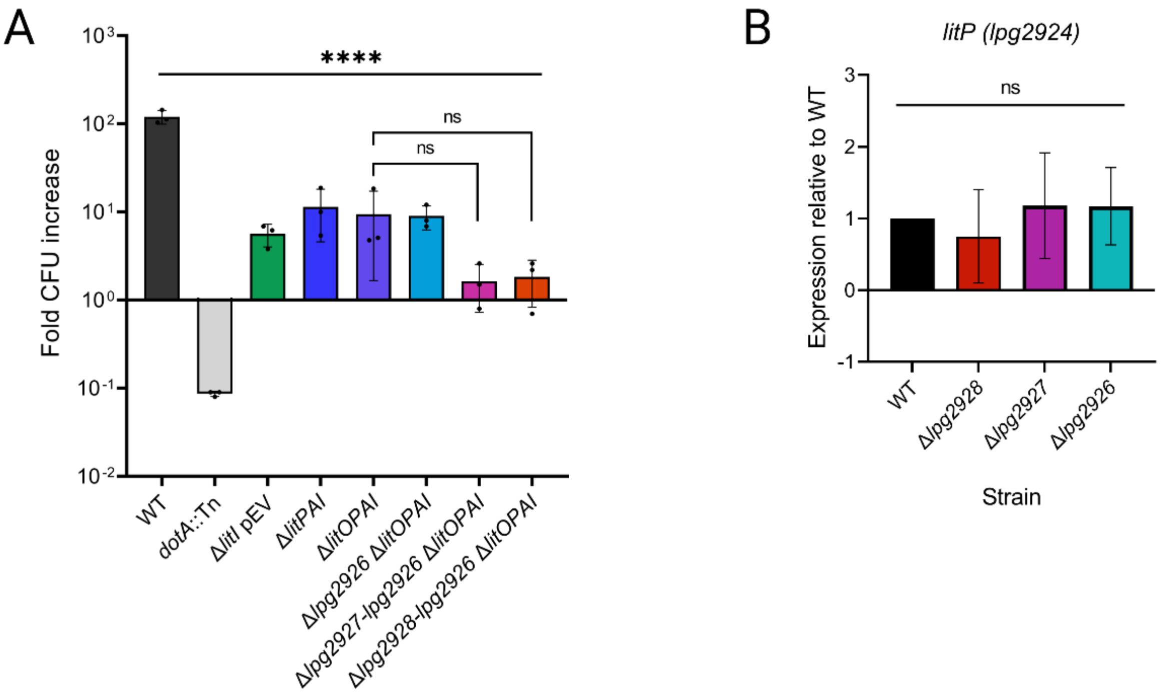

Figure S8. Examination of a potential functional relationship between LitOPAI and Lpg2928-Lpg2926. (A) Fold change in CFUs of multi-gene deletion mutants in A/J BMDMs over 72h. Asterisks indicate statistical significance by one-way ANOVA (****p<0.0001). Results are representative of at least two independent experiments. **(B)** Relative expression of *litP* in deletion mutants compared to WT. Expression levels are normalized to expression of *gyrB*. Ns; not significant.

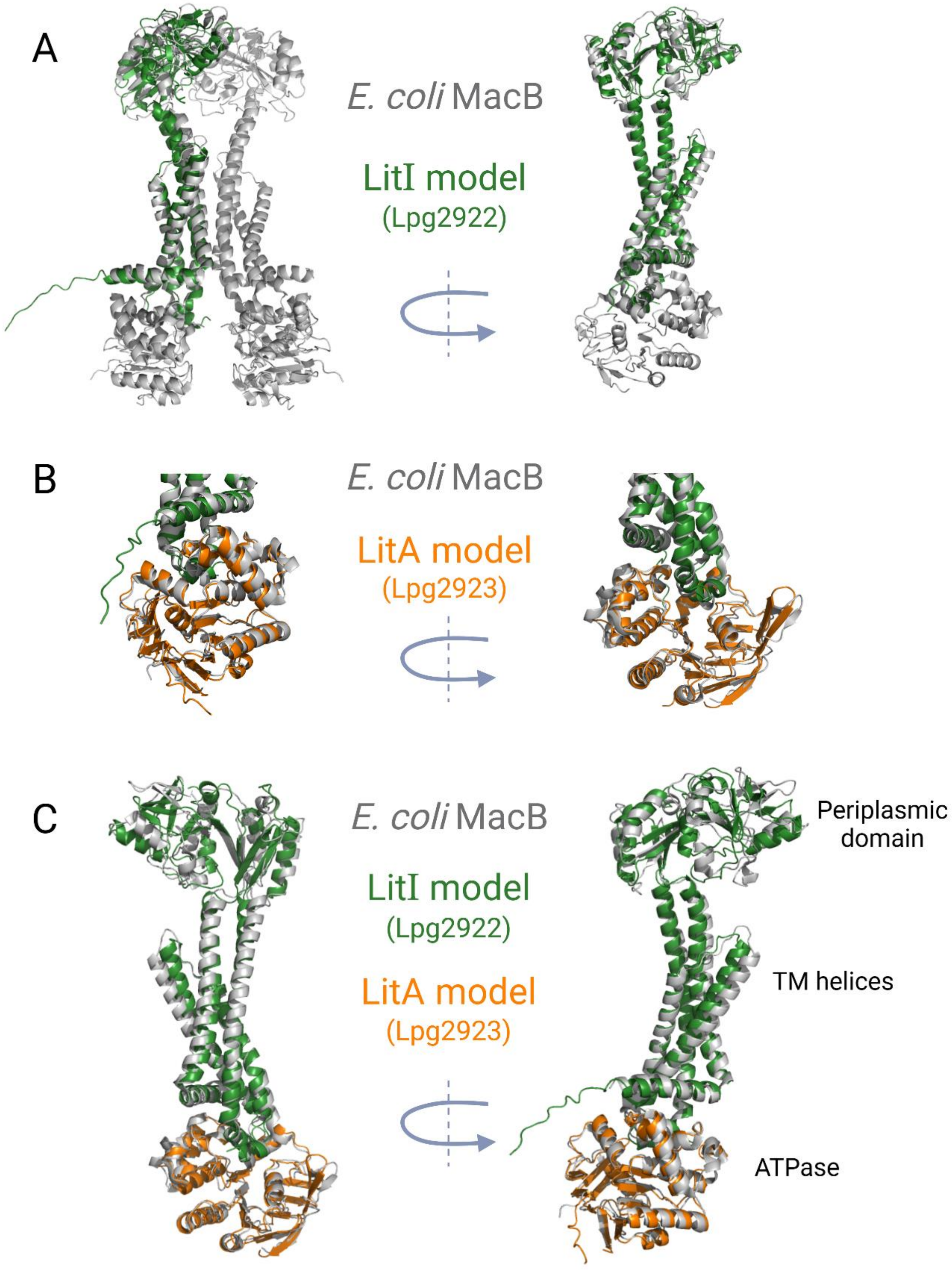

Figure S9. LitI and LitA have strong predicted structural homology to MacB. ColabFold structural models of LitI (Lpg2922) and LitA (Lpg2923) were aligned with the structure of MacB from *E. coli* (29) (PDB ID: 5NIK) using the PyMOL Molecular Graphics System (14);

RMSD 3.45. **(A)** Model of LitI permease (green) aligned with MacB (grey). Left: Dimer of MacB aligned with monomer of LitI. Right: Monomers of MacB and LitI aligned and rotated. **(B)** Model of LitA ATPase (orange) aligned with lower domain of MacB; RMSD 1.23. **(C)** Models of LitI and LitA aligned to MacB structure. TM; transmembrane. Created using BioRender.com.

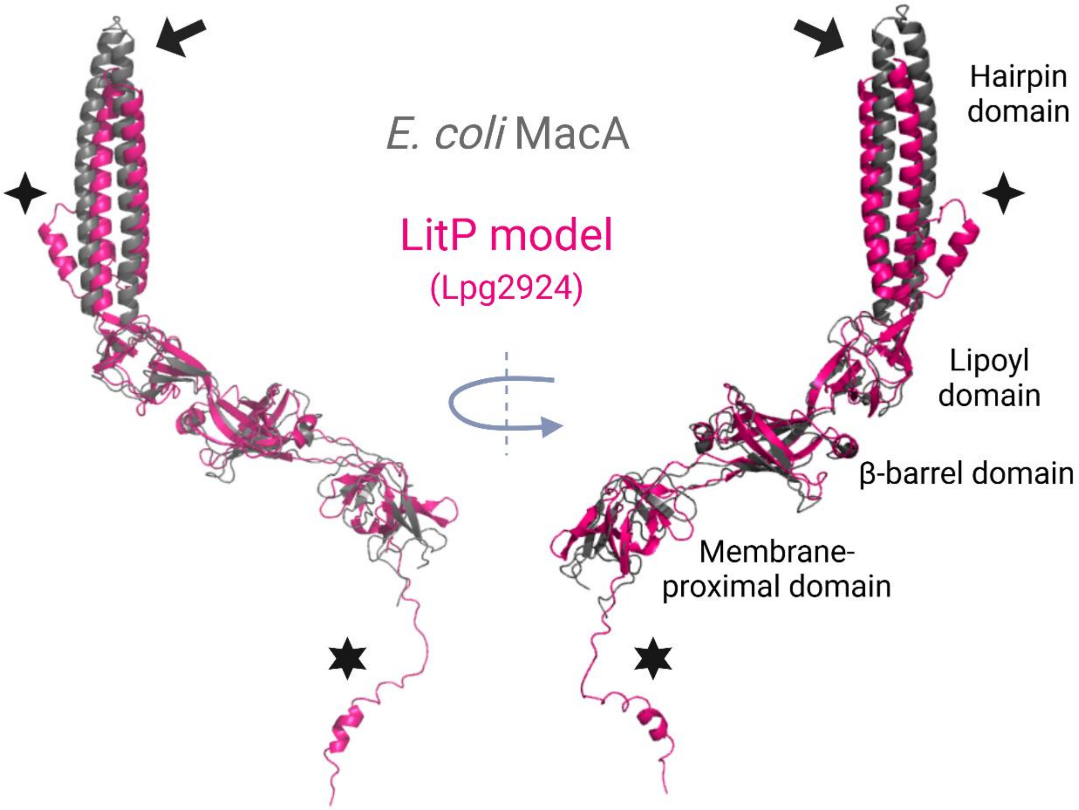

Figure S10. LitP has predicted structural homology to MacA. A ColabFold structural model of LitP (Lpg2924; magenta) was aligned with the structure of MacA from *E. coli* (grey) (29) (PDB ID: 5NIK) using the PyMOL Molecular Graphics System (14); RMSD 4.65. Domains are labelled on the right. Arrows; shortened hairpin domain. Four-pointed stars; additional helices. Six-pointed stars: note that the N-terminus of MacA is missing from the structure, likely due to flexibility in this region (29). Created using BioRender.com.

REFERENCES: Supplemental information

1. **Goodman AL, McNulty NP, Zhao Y, Leip D, Mitra RD, Lozupone CA, Knight R, Gordon JI.** 2009. Identifying genetic determinants needed to establish a human gut symbiont in its habitat. Cell Host Microbe **6:**279-289.

2. **Shames SR, Liu L, Havey JC, Schofield WB, Goodman AL, Roy CR.** 2017. Multiple Legionella pneumophila effector virulence phenotypes revealed through high-throughput analysis of targeted mutant libraries. Proc Natl Acad Sci U S A **114:**E10446-E10454.

3. **Goodman AL, Wu M, Gordon JI.** 2011. Identifying microbial fitness determinants by insertion sequencing using genome-wide transposon mutant libraries. Nat Protoc **6:**1969-1980.

4. **Tatusov RL, Koonin EV, Lipman DJ.** 1997. A genomic perspective on protein families. Science **278:**631-637.

5. **Galperin MY, Wolf YI, Makarova KS, Vera Alvarez R, Landsman D, Koonin EV.** 2021. COG database update: focus on microbial diversity, model organisms, and widespread pathogens. Nucleic Acids Res **49:**D274-D281.

6. **Hildebrand A, Remmert M, Biegert A, Soding J.** 2009. Fast and accurate automatic structure prediction with HHpred. Proteins **77:**128-132.

7. **Zimmermann L, Stephens A, Nam S, Rau D, Kubler J, Lozajic M, Gabler F, Soding J, Lupas AN, Alva V.** 2018. A completely reimplemented MPI Bioinformatics Toolkit with a new HHpred server at its core. J Mol Biol **430:**2237-2243.

8. **Gabler F, Nam S, Till S, Mirdita M, Steinegger M, Soding J, Lupas AN, Alva V.** 2020. Protein sequence analysis using the MPI Bioinfomatics Toolkit. Curr Protoc Immunol **72**.

9. **Altschul SF, Gish W, Miller W, Myers EW, Lipman DJ.** 1990. Basic local alignment search tool. J Mol Biol **215:**403-410.

10. **Mirdita M, Schutze K, Moriwaki Y, Heo L, Ovchinnikov S, Steinegger M.** 2022. ColabFold: making protein folding accessible to all. Nat Methods **19:**679-682.

11. **Jumper J, Evans R, Pritzel A, Green T, Figurnov M, Ronneberger O, Tunyasuvunakool K, Bates R, Zidek A, Potapenko A, Bridgland A, Meyer C, Kohl SAA, Ballard AJ, Cowie A, Romera-Paredes B, Nikolov S, Jain R, Adler J, Back T, Petersen S, Reiman D, Clancy E, Zielinski M, Steinegger M, Pacholska M, Berghammer T, Bodenstein S, Silver D, Vinyals O, Senior AW, Kavukcuoglu K, Kohli P, Hassabis D.** 2021. Highly accurate protein structure prediction with AlphaFold. Nature **596:**583-589.

12. **Varadi M, Anyango S, Deshpande M, Nair S, Natassia C, Yordanova G, Yuan D, Stroe O, Wood G, Laydon A, Zidek A, Green T, Tunyasuvunakool K, Petersen S, Jumper J, Clancy E, Green R, Vora A, Lutfi M, Figurnov M, Cowie A, Hobbs N, Kohli P, Kleywegt G, Birney E, Hassabis D, Velankar S.** 2021. AlphaFold protein structure database: massively expanding the structural coverage of protein-sequence space with high-accurary models. Nucleic Acids Res **50:**D439-D444.

13. **Steinegger M, Soding J.** 2017. MMseqs2 enables sensitive protein sequence searching for the analysis of massive data sets. Nat Biotechnol **35:**1026-1028.

14. **Schrodinger, LLC.** 2015. The PyMOL Molecular Graphics System, Version 2.6.0a0.

15. **Zuckman DM, Hung JB, Roy CR.** 1999. Pore-forming activity is not sufficient for *Legionella pneumophila* phagosome trafficking and intracellular growth. Mol Microbiol **32:**990-1001.

16. **Nagai H, Roy CR.** 2001. The DotA protein from *Legionella pneumophila* is secreted by a novel process that requires the Dot/Icm transporter. the EMBO Journal **20:**5962-5970.

17. **Creasey EA, Isberg RR.** 2012. The protein SdhA maintains the integrity of the Legionella-containing vacuole. Proc Natl Acad Sci U S A **109:**3481-3486.

18. **Laguna RK, Creasey EA, Li Z, Valtz N, Isberg RR.** 2006. A *Legionella pneumophila*-translocated substrate that is required for growth within macrophages and protection from host cell death. Proc Natl Acad Sci U S A **103:**18745-18750.

19. **Berger KH, Isberg RR.** 1993. Two distinct defects in intracellular growth complemented by a single genetic locus in Legionella pneumophila. Mol Microbiol **7:**7-19.

20. **Segal G, Purcell M, Shuman HA.** 1998. Host cell killing and bacterial conjugation require overlapping sets of genes within a 22-kb region of the Legionella pneumophila genome. Proc Natl Acad Sci U S A **95:**1669-1674.

21. **Edelstein PH, Hu B, Higa F, Edelstein MAC.** 2003. *lvgA*, a novel *Legionella pneumophila* virulence factor. Infect Immun **71:**2394-2403.

22. **Kim H, Kubori T, Yamazaki K, Kwak M, Park S, Nagai H, Vogel JP, Oh B.** 2020. Structural basis for effector protein recognition by the Dot/Icm Type IVB coupling protein complex. Nat Commun **11**.

23. **Sauer J, Bachman MA, Swanson MS.** 2005. The phagosomal transporter A couples threonine acquisition to differentiation and replication of Legionella pneumophila in macrophages. Proc Natl Acad Sci U S A **102:**9924–9929.

24. **Ferhat M, Atlan D, Vianney A, Lazzaroni JC, Doublet P, Gilbert C.** 2009. The TolC protein of Legionella pneumophila plays a major role in multi-drug resistance and the early steps of host invasion. PLoS One **4:**e7732.

25. **Rossier O, Starkenburg SR, Cianciotto NP.** 2004. *Legionella pneumophila* Type II protein secretion promotes virulence in the A/J mouse model of Legionnaires' disease pneumonia. Infect Immun **72:**310-321.

26. **Miyake M, Watanabe T, Koike H, Molmeret M, Imai Y, Abu Kwaik Y.** 2005. Characterization of *Legionella pneumophila pmiA*, a gene essential for infectivity of protozoa and macrophages. Infect Immun **73:**6272-6282.

27. **Joseph AM, Pohl AE, Ball TJ, Abram TG, Johnson DK, Geisbrecht BV, Shames SR.** 2020. The Legionella pneumophila Metaeffector Lpg2505 (MesI) Regulates SidI-Mediated Translation Inhibition and Novel Glycosyl Hydrolase Activity. Infect Immun **88**.

28. **Isaac DT, Laguna RK, Valtz N, Isberg RR.** 2015. MavN is a Legionella pneumophila vacuole-associated protein required for efficient iron acquisition during intracellular growth. Proc Natl Acad Sci U S A **112:**E5208-5217.

29. **Fitzpatrick AWP, Llabres S, Neuberger A, Blaza JN, Bai XC, Okada U, Murakami S, van Veen HW, Zachariae U, Scheres SHW, Luisi BF, Du D.** 2017. Structure of the MacAB-TolC ABC-type tripartite multidrug efflux pump. Nat Microbiol **2:**17070.
